# Substrate Specificities and Inhibition Pattern of the Solute Carrier Family 10 Members NTCP, ASBT and SOAT

**DOI:** 10.3389/fmolb.2021.689757

**Published:** 2021-05-17

**Authors:** Gary Grosser, Simon Franz Müller, Michael Kirstgen, Barbara Döring, Joachim Geyer

**Affiliations:** Institute of Pharmacology and Toxicology, Faculty of Veterinary Medicine, Justus Liebig University Giessen, Biomedical Research Center Seltersberg (BFS), Giessen, Germany

**Keywords:** SLC10A1, SLC10A2, substrate specificity, drug target, NTCP, transport inhibitor, cross-reactivity, SLC10A6

## Abstract

Three carriers of the solute carrier family SLC10 have been functionally characterized so far. Na^+^/taurocholate cotransporting polypeptide NTCP is a hepatic bile acid transporter and the cellular entry receptor for the hepatitis B and D viruses. Its intestinal counterpart, apical sodium-dependent bile acid transporter ASBT, is responsible for the reabsorption of bile acids from the intestinal lumen. In addition, sodium-dependent organic anion transporter SOAT specifically transports sulfated steroid hormones, but not bile acids. All three carriers show high sequence homology, but significant differences in substrate recognition that makes a systematic structure-activity comparison attractive in order to define the protein domains involved in substrate binding and transport. By using stably transfected NTCP-, ASBT-, and SOAT-HEK293 cells, systematic comparative transport and inhibition experiments were performed with more than 20 bile acid and steroid substrates as well as different inhibitors. Taurolithocholic acid (TLC) was identified as the first common substrate of NTCP, ASBT and SOAT with *K*_m_ values of 18.4, 5.9, and 19.3 µM, respectively. In contrast, lithocholic acid was the only bile acid that was not transported by any of these carriers. Troglitazone, BSP and erythrosine B were identified as pan-SLC10 inhibitors, whereas cyclosporine A, irbesartan, ginkgolic acid 17:1, and betulinic acid only inhibited NTCP and SOAT, but not ASBT. The HBV/HDV-derived myr-preS1 peptide showed equipotent inhibition of the NTCP-mediated substrate transport of taurocholic acid (TC), dehydroepiandrosterone sulfate (DHEAS), and TLC with IC_50_ values of 182 nM, 167 nM, and 316 nM, respectively. In contrast, TLC was more potent to inhibit myr-preS1 peptide binding to NTCP with IC_50_ of 4.3 µM compared to TC (IC_50_ = 70.4 µM) and DHEAS (IC_50_ = 52.0 µM). Based on the data of the present study, we propose several overlapping, but differently active binding sites for substrates and inhibitors in the carriers NTCP, ASBT, SOAT.

## Introduction

The solute carrier family SLC10, also known as the “sodium bile acid cotransporter family” currently consists of seven members (SLC10A1-SLC10A7) (Geyer et al., 2006; Claro da Silva et al., 2013). Three of them (SLC10A1, SLC10A2, and SLC10A6) have been functionally characterized, while the members SLC10A3, SLC10A4, SLC10A5, and SLC10A7 are still orphan carriers (Fernandes et al., 2007; Geyer et al., 2007; Godoy et al., 2007; Karakus et al., 2020). The founding members of the SLC10 carrier family were cloned in the early 1990s and were termed Na^+^/taurocholate cotransporting polypeptide (NTCP, gene symbol *SLC10A1*) (Hagenbuch and Meier, 1994) and apical sodium-dependent bile acid transporter (ASBT, gene symbol *SLC10A2*) (Wong et al., 1996), both sharing 39% amino acid sequence identity. NTCP is exclusively expressed at the basolateral (sinusoidal) membrane of hepatocytes (Ananthanarayanan et al., 1994; Stieger et al., 1994) and here mediates sodium-coupled uptake of taurocholic acid (TC) and other bile acids (BA) with a Na^+^:BA stoichiometry of 2:1 (Hagenbuch and Meier, 1996; Weinman, 1997). ASBT is typically expressed in the apical brush border membrane of enterocytes of the terminal ileum (Shneider et al., 1995), where it transports conjugated BAs with high affinity in a sodium-dependent manner (Craddock et al., 1998). Both carriers are essentially involved in the maintenance of the enterohepatic circulation of BAs (Döring et al., 2012). In 2007, we cloned an additional SLC10 carrier, named sodium-dependent organic anion transporter (SOAT, gene symbol *SLC10A6*) (Geyer et al., 2007). Although SOAT shows the highest sequence identity of 48% to ASBT, it does not represent a BA transporter (Geyer et al., 2007). In contrast, SOAT specifically transports 3′ sulfated steroid hormones such as estrone-3-sulfate (E_1_S), estradiol-3-sulfate, dehydroepiandrosterone sulfate (DHEAS), androstenediol-3-sulfate, androsterone-3-sulfate, and pregnenolone sulfate (PREGS) (Fietz et al., 2013) and, thereby, has a role for steroid supply to different organs (Geyer et al., 2017). In addition, SOAT transports 17′ sulfated steroids such as testosterone-17β-sulfates, but not steroid disulfates such as 17β-estradiol-3,17-disulfate (Grosser et al., 2018).

Apart from their roles as physiological uptake carriers for BAs and sulfated steroid hormones, all three carriers were also established as drug targets. In 2012, NTCP was identified as the high-affinity hepatic entry receptor for the hepatitis B (HBV) and hepatitis D (HDV) viruses (Yan et al., 2012; Drexler et al., 2013). More precisely, both viruses bind to NTCP with their 2–48 N-terminal amino acids of the myristoylated preS1 domain (so-called myr-preS1 peptide) of the large envelope protein and this triggers the cellular entry of the virus/NTCP complex (Iwamoto et al., 2019). Interestingly, BA binding and myr-preS1 peptide binding to NTCP directly interfere with each other. BAs can block myr-preS1 peptide binding to NTCP and *in vitro* HBV/HDV infection, while myr-preS1 peptide binding to NTCP inhibits BA transport (König et al., 2014; Ni et al., 2014). Apart from the myr-preS1 peptide, several small molecules were detected that also block virus binding to NTCP *in vitro*, such as cyclosporine A, ezetimibe, irbesartan, ritonavir, troglitazone or betulinic acid (Fukano et al., 2019; Kirstgen et al., 2020; Li et al., 2020; Wettengel and Burwitz, 2020), but none of them is clinically approved for HBV/HDV entry inhibition yet. In contrast, pharmacological inhibitors of ASBT, such as odevixibat and maralixibat, are already in clinical use. These so-called bile acid reabsorption inhibitors (BARIs) are used to treat BA-related diseases such as intrahepatic cholestasis, primary biliary cholangitis, Alagille syndrome, or non-alcoholic steatohepatitis (Karpen et al., 2020). In addition, BARIs are used to treat chronic constipation by increasing the intestinal BA content and to lower plasma LDL-cholesterol levels by increasing the *de novo* hepatic synthesis of BAs from the precursor cholesterol (Kramer and Glombik, 2006; Al-Dury and Marschall, 2018). SOAT is expressed in breast cancer and here mediates the uptake of pro-proliferative sulfated estrogen precursors. Inhibition of SOAT had anti-proliferative effects in breast cancer cells *in vitro*, and so was proposed as potential novel anti-cancer drug target (Karakus et al., 2018).

Since the cloning of SOAT it is still an open question why the close phylogenetic relationship of ASBT and SOAT is not reflected at the functional level, while the more distant carriers NTCP and ASBT are close functional homologs. Therefore, in the present study we aimed to compare systematically the substrate specificities of NTCP, ASBT and SOAT and their inhibition pattern. Based on the data of the present study, we propose several overlapping substrate and inhibitor binding sites at the three carriers that have to be considered as potential off-target sites when one of these carriers is addressed with pharmacological inhibitors. Furthermore, we identified taurolithocholic acid (TLC) as the first common substrate of all three carriers.

## Materials and Methods

### Radiochemicals and Chemicals

[^3^H]Dehydroepiandrosterone sulfate ([^3^H]DHEAS), [^3^H]estrone-3-sulfate ([^3^H]E_1_S), [^3^H]cortisone, [^3^H]pregnenolone sulfate ([^3^H]PREGS), [^3^H]chenodeoxycholic acid, [^3^H]lithocholic acid and [^3^H]taurocholic acid ([^3^H]TC) were imported via BIOTREND Chemikalien GmbH (Cologne, Germany) from the manufacturer American Radiolabeled Chemicals, Inc. (St. Louis, United States). [^3^H]Cortisol was obtained from Perkin Elmer, Inc. (Boston, United States). [^3^H]Estrone-3β**-**D-glucuronide and [^3^H]estradiol-17β**-**D-glucuronide were generously provided by Dr. Bernhard Ugele (Munich, Germany). [^3^H]Cholic acid, [^3^H]deoxycholic acid, [^3^H]ursodeoxycholic acid, [^3^H]sarcosine cholic acid, [^3^H]glycodeoxycholic acid, [^3^H]glycochenodeoxycholic acid, [^3^H]glycoursodeoxycholic acid, [^3^H]taurodeoxycholic acid, [^3^H] tauroursodeoxycholic acid and [^3^H]taurochenodeoxycholic acid were generously provided by Prof. Dr. Alan Hofmann, University of California (San Diego, United States). [^3^H]Taurolithocholic acid ([^3^H]TLC) was synthesized as described before (Lowjaga et al., 2021).

Estrone-3-sulfate (E_1_S), pregnenolone sulfate (PREGS), dehydroepiandrosterone sulfate (DHEAS), and taurocholic acid (TC) were obtained from Sigma-Aldrich (St. Louis, United States). Zeocin and hygromycin were purchased from Invitrogen (Groningen, Netherlands). A set of betulin derivatives (betulin, betulinic acid, lupenone, 3-O-caffeoyl betulin) was purchased from Adipogen AG (Liestal, Switzerland). Ezetimibe, bromosulfophthalein (BSP), irbesartan, losartan, erythrosine B, and ginkgolic acid C17:1, and all other chemicals if not stated otherwise were purchased from Sigma-Aldrich (St. Louis, United States). Cyclosporine A was purchased from Tokyo Chemical Industry (Tokyo, Japan). Troglitazone was purchased from Cayman Chemical (Michigan, United States).

### NTCP-HEK293, ASBT-HEK293, and SOAT-HEK293 Cells

The full-length open reading frames of NTCP, ASBT, and SOAT were cloned based on the cDNA sequences with GenBank accession numbers NM_003049 (NTCP), NM_000452 (ASBT) and NM_197965 (SOAT), respectively, as reported before (Geyer et al., 2007; Grosser et al., 2015). Sequence verified clones were used for stable transfection of Flp-In T-REX HEK293 cells (HEK293-FlpIn) according to the manufacturer’s instructions (Invitrogen) as reported (Geyer et al., 2007). From the generated NTCP-HEK293, ASBT-HEK293, and SOAT-HEK293 cells transgene expression can be induced by tetracycline treatment. Cells were maintained under D-MEM/F12 medium supplemented with 10% fetal calf serum (Sigma-Aldrich), l-glutamine (4 mM), penicillin (100 U/ml), and streptomycin (100 μg/ml) (further referred to as standard medium) at 37°C, 5% CO_2_, and 95% humidity. All cell culture materials and substances were purchased from Thermo Fisher Scientific (Waltham, United States) if not stated otherwise.

### Cultivation and Induction of Stably Transfected HEK293 Cells for Uptake, Inhibition or Binding Studies

Stably SLC10 transporter transfected NTCP-HEK293, ASBT-HEK293, SOAT-HEK293 cells and the HEK293-FlpIn maternal cell line were seeded on 24-well plates (if not stated otherwise for individual assays). Well plates were coated with poly-d-lysine prior to seeding of 125,000 cells per well. Cells were grown with 1 ml of standard medium per well with or without tetracycline (1 mg/ml) for 72 h to induce carrier expression before respective assays were started. HEK293-FlpIn cells were cultivated with standard medium and served as control.

### Substrate Screening in NTCP-HEK293, ASBT-HEK293, SOAT-HEK293, and HEK293-FlpIn Cells

Cells were washed three times with phosphate buffered saline (PBS) (137 mM NaCl, 2.7 mM KCl, 1.5 mM KH_2_PO_4_, 7.3 mM Na_2_HPO_4_, pH 7.4, 37°C). Afterward, cells were preincubated at 37°C with sodium transport buffer (containing 142.9 mM NaCl, 4.7 mM KCl, 1.2 mM MgSO_4_, 1.2 mM KH_2_PO_4_, 1.8 mM CaCl_2_, and 20 mM HEPES (all chemicals from Sigma-Aldrich), adjusted to pH 7.4), or with choline transport buffer (equimolar substitution of sodium chloride with choline chloride). Uptake experiments were initiated by replacing the preincubation buffer by 500 μL transport buffer containing the radiolabeled test compound and were performed at 37°C. Transport was terminated by removing the transport buffer and washing five-times with ice-cold PBS. Cell monolayers were lysed in 1 N NaOH with 0.1% SDS and the cell-associated radioactivity was determined by liquid scintillation counting. Protein content of individual wells was determined by Lowry assay as reported before (Geyer et al., 2007).

### Transport Inhibition in NTCP-HEK293, ASBT-HEK293, SOAT-HEK293, and HEK293-FlpIn Cells

Cells were washed three times with PBS and were preincubated with the respective inhibitor in sodium transport buffer for 5 min at 37°C. Uptake was initiated by adding the respective radiolabeled substrate to the well and incubating for a fixed time as indicated in the figures at 37°C. Transport was terminated by removing the transport buffer and washing five-times with ice-cold PBS. Cell monolayers were lysed in 1 N NaOH with 0.1% SDS and the cell-associated radioactivity and protein content was determined as described above. For uptake inhibition with the myr-preS1 peptide the sodium transport buffer contained additionally MEM-amino acid solution (ThermoFisher) at 1:50 dilution.

### Binding Assays With the Myr-preS1 Peptide

NTCP-HEK293 cells were seeded into 24-well-dishes as described above. For every set of induced wells an equal number of not-induced wells were used as respective background controls. Cells were washed three times with PBS and then preincubated with sodium transport buffer supplemented with MEM-amino acid solution (ThermoFisher) at 1:50 dilution at 37°C for 5 min. The fluorescent myr-preS1-Al633 peptide was added with a final concentration of 10 nM and binding experiments were performed over 10 min at 37°C. Then, cells were washed twice with buffer at 37°C and transferred to the fluorescence reader Typhoon (GE Healthcare, Chicago, United States) to quantitatively determine bound fluorescence signals as established in our lab before (Müller et al., 2018). For calculation of the NTCP-specific binding signal, the mean background signal from the not-induced cells was subtracted. Net binding rates in the absence of any inhibitor were set to 100%.

### Phylogenetic Analysis

Phylogenetic analysis of the SLC10 carriers was performed using the proteins with the following GenBank accession numbers. NP_003040.1 for NTCP/SLC10A1, NP_000443.2 for ASBT/SLC10A2, NP_689892.1 for SLC10A3, NP_689892.1 for SLC10A4, NP_001010893.1 for SLC10A5, NP_932069.1 for SOAT/SLC10A6, AAI50309.1 for SLC10A7, and O15245.2 for OCT1 as outroot. In addition, the bacterial proteins Asbt_Nm_ (PDB: 3ZUY.A) and Abst_Yf_ (PDB: 4N7X.A) were included. The phylogenetic tree was generated based on sequence alignment ClustalW (Lasergene DNASTAR) and was visualized with the FigTree tool (tree.bio.ed.ac.uk). Scale bar represents 0.1 changes per site on horizontal distance.

### Quantitative Real Time PCR of Transporter Expression

The mRNA expression pattern of NTCP, ASBT and SOAT in the NTCP-HEK293, ASBT-HEK293, SOAT-HEK293, and HEK293-FlpIn cells was analyzed by quantitative real-time PCR with cDNA from the indicated tetracycline induced stably transfected cell lines. RNA was isolated from the respective stably transfected cells or control cells grown in 6 well plates following 72 h of growth. The medium and any detached cells were removed from the well. Total RNA isolation was performed by using peqGOLD RNAPure reagent (PeqLab, Erlangen, Germany) according to the manufacturer’s protocol. The RNA concentration was determined by measuring absorbance at 260 nm with a Beckmann spectrophotometer DU-640 (Beckmann, Munich, Germany). Complementary cDNA was synthesized from the RNA samples using SuperScript III First-Strand Synthesis System for RT-PCR according to the manufacturer’s protocol (Invitrogen, Karlsruhe). Relative carrier expression was calculated by the 2^−ΔΔCT^ method and represents carrier expression x-times higher compared with the calibrator (NTCP expression in SOAT-HEK293 cells). ACTB served as endogenous control. Values represent means of duplicate determinations. Relative gene expression analysis was performed by real-time PCR amplification on an ABI PRISM 7300 thermal cycler (Applied Biosystems, Darmstadt, Germany) using the TaqMan Gene Expression Assays Hs01399354_m1 for SOAT, Hs00166561_m1 for ASBT, Hs00161820_m1 for NTCP, and Hs99999903_m1 for ACTB (Applied Biosystems, Darmstadt, Germany). Real-time amplification was performed in 96-well optical plates using 5 µL cDNA, 1.25 µL TaqMan Gene Expression Assay, 12.5 µL TaqMan universal PCR Master Mix and 6.25 µL water in each 25 µL reaction. The plates were heated for 10 min at 95°C, and 45 cycles of 15 s at 95°C and 60 s at 60°C were applied.

### Data Analysis and Statistics

All transport or inhibition graphs were generated with GraphPad Prism 6.0 (GraphPad). Determination of IC_50_ values was done by nonlinear regression analysis using the equation log (inhibitor) vs. response settings. If not stated otherwise in the legends all data represent means ± SD of at least triplicate determinations of representative experiments.

### In silico Docking

The crystal structures of two bacterial SLC10-homologous carriers have been published, namely Asbt from *Neisseria meningitidis* (Asbt_Nm_) and Asbt from *Yersinia frederiksenii* (Asbt_Yf_) (Hu et al., 2011; Zhou et al., 2014). Based on a more recent publication that verified the crystal structure of Abst_Yf_ (4n7x.1.a) as an outward facing model for BA transporters (Wang et al., 2021) we generated outward facing homology models of NTCP, ASBT and SOAT based on this structure via the SWISS-MODEL server (https://swissmodel.expasy.org). These models were used as input structures in SwissDock (http://www.swissdock.ch/docking) and were *in silico* docked with TLC with standard parameters. The obtained docked clusters and models were visualized with the UCSF CHIMERA software (https://www.cgl.ucsf.edu/chimera/). For visualization, docked clusters were reduced to TLC molecules in reasonable proximity to the putative outward facing binding pocket.

### Pharmacophore Calculation

Generation of pharmacophore models was performed using PHASE (Dixon et al., 2006), integrated into the MAESTRO Molecular Modeling Interface (Version 12.2) of SCHRÖDINGER, Inc. (www.schrodinger.com, New York City, NY, United States). The following settings were used. Active/inactive (see Table 1), hypothesis should match at least 50% of actives, 4–5 features in the hypothesis, difference criterion 0.5 , create excluded volume shell from actives and inactives, minimum number of inactives that must experience a clash = 1, minimum distance between active surface and excluded volumes 1 Å, excluded volume sphere radii 1 Å.

**TABLE 1:** Substrate specificities and transport activity grading for NTCP, ASBT and SOAT. The primary transport data are indicated in Figure 1. The following grading was used. “—” represents no significant uptake compared to sodium-free control. Mean uptake “+” for values below 10, “++” for values between 10 and 100, and “+++” for values above 100 pmol/mg protein/10 min for sulfated steroid hormones. Mean uptake of bile acids “+” for values below 500, “++” for values between 500 and 1000, and “+++” for values above 1000 pmol/mg protein/30 min.

	NTCP	ASBT	SOAT
Cholic acid	++	+	—
Chenodeoxycholic acid	+	+	—
Deoxycholic acid	+	+	—
Ursodeoxycholic acid	+	—	—
Lithocholic acid	—	—	—
Sarcosine cholic acid	+	+	—
Glycocholic acid	+++	++	—
Glycochenodeoxycholic acid	+++	++	—
Glycodeoxycholic acid	+++	++	—
Glycoursodeoxycholic acid	+++	++	—
Taurocholic acid	+++	++	—
Taurochenodeoxycholic acid	+++	++	—
Taurodeoxycholic acid	+++	++	—
Tauroursodeoxycholic acid	+++	++	—
Taurolithocholic acid	+++	+++	++
DHEAS	+	—	++
E_1_S	++	—	+
PREGS	+++	—	+++
Estrone-3β-D-glucuronide	+	—	—
Estradiol-17β-D-glucuronide	—	—	—
Cortisone	—	—	—
Cortisol	—	—	—

## Results

### Comparative Substrate Screening for NTCP, ASBT, and SOAT

This study focused explicitly on comparative transport studies with NTCP, ASBT and SOAT. Therefore, stably transfected HEK293 cells were generated based on the identical HEK293-FlpIn cell line and following the identical protocol. The generated cell lines NTCP-HEK293, ASBT-HEK293, and SOAT-HEK293 showed significant overexpression of the respective carrier as shown by comparative quantitative expression analysis (see Supplementary Figure S1).

**FIGURE 1:**
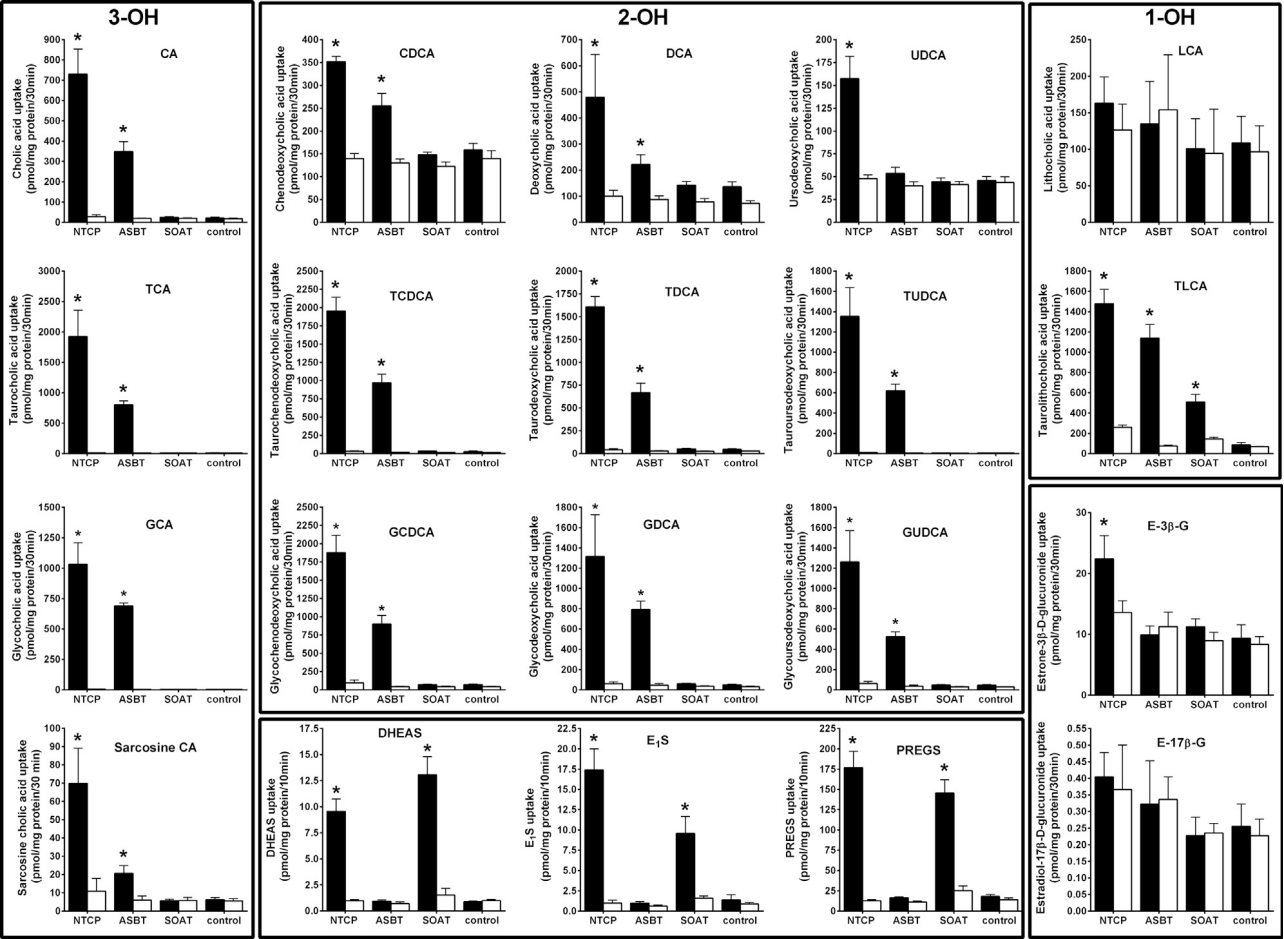
Comparative substrate screening for bile acids and sulfated steroids in NTCP-, ASBT-, and SOAT-HEK293 cells. Transport assays were performed with the indicated substrates in NTCP-HEK293, ABST-HEK293, SOAT-HEK293, and HEK293-FlpIn cells. Cells were washed and equilibrated with Na^+^-containing transport buffer (filled bars) or with Na^+^-free transport buffer (open bars) at 37°C. For uptake, cells were incubated with radiolabeled steroid compounds at a final concentration of 200 nM in Na^+^-containing or Na^+^-free transport buffer for 10 min. Radiolabeled bile acids were used at 1 µM concentrations and transport was analyzed over 30 min. HEK293-FlpIn cells served as additional controls. After the indicated time intervals, cells were washed with ice-cold PBS, lysed and subjected to liquid scintillation counting. Values represent means ± SD of combined data of two independent experiments, each with quadruplicate determinations (*n* = 8). *p < 0.01 (two-way ANOVA) indicating Na^+^-dependent uptake (Na^+^-containing vs. Na^+^-free transport buffer of the respective cell line). 3-OH, trihydroxylated BAs; 2-OH, dihydroxylated BAs; 1-OH, monohydroxylated BAs.

In a first approach, these cell lines were used to analyze the sodium-dependent uptake of different BAs and steroid derivatives (Figure 1). Therefore, all transport studies were performed in sodium-containing transport buffer (filled bars) as well as in sodium-free transport buffer (open bars). The non-transfected HEK293-FlpIn cell line served as additional control. In detail, this panel of test compounds included different unconjugated BAs as well as glycine-conjugated and taurine-conjugated BAs. All BAs derived from cholic acid can be classified as 3α,7α,12α-trihydroxylated (3-OH) BAs. Together with the 3α,7α-dihydroxylated (2-OH) chenodeoxycholic acid-derived BAs, these are primary BAs synthesized in the liver. Two other groups of dihydroxylated BAs, namely deoxycholic acid and ursodeoxycholic acid result from bacterial de-conjugation and isomerization, respectively. These are classified as secondary BAs. In addition, the 3α-monohydroxylated (1-OH) BA lithocholic acid results from bacterial de-conjugation in the gut. Secondary BAs are mostly reabsorbed in their unconjugated forms and then can be reconjugated in the liver with glycine or taurine. As an additional BA derivative, sarcosine cholic acid was included. From the group of steroid conjugates, three sulfo-conjugated steroids, namely E_1_S, DHEAS, and PREGS were analyzed, as well as the steroid glucuronides estrone-3β-D-glucuronide and estradiol-17β-D-glucuronide. Finally, the glucocorticoids cortisone and cortisol were used for transport experiments.

All primary transport data are presented in Figure 1. Additionally, the primary transport data were arranged in a graded manner in Table 1 for better overview and comparability. As expected, NTCP and ASBT showed significant and sodium-dependent transport activity for nearly all BAs analyzed. The transport rates of taurine- and glycine-conjugated BAs by ASBT and NTCP are obviously higher compared to the unconjugated forms. Among the group of unconjugated BAs, ursodeoxycholic acid was only transported by NTCP, but not by ASBT, and lithocholic acid was the only BA that was transported neither by NTCP nor by ASBT. Of note, also the BA derivative sarcosine cholic acid was significantly transported by NTCP and ASBT in a sodium-dependent manner. Apart from the group of BAs, NTCP showed also significant transport activity for the steroid conjugates DHEAS, E_1_S, PREGS, and estrone-3β-D-glucuronide, but not for estradiol-17β-D-glucuronide. The steroid sulfate carrier SOAT showed significant transport activity for DHEAS, E_1_S, and PREGS as expected, but was transport negative for the steroid glucuronides and for nearly all BAs. Surprisingly, SOAT showed significant sodium-dependent uptake of TLC and, therefore, TLC is the only common substrate of all three carriers NTCP, ASBT and SOAT, identified so far.

### Comparative Transport Kinetics for NTCP, ASBT, and SOAT

**FIGURE 2:**
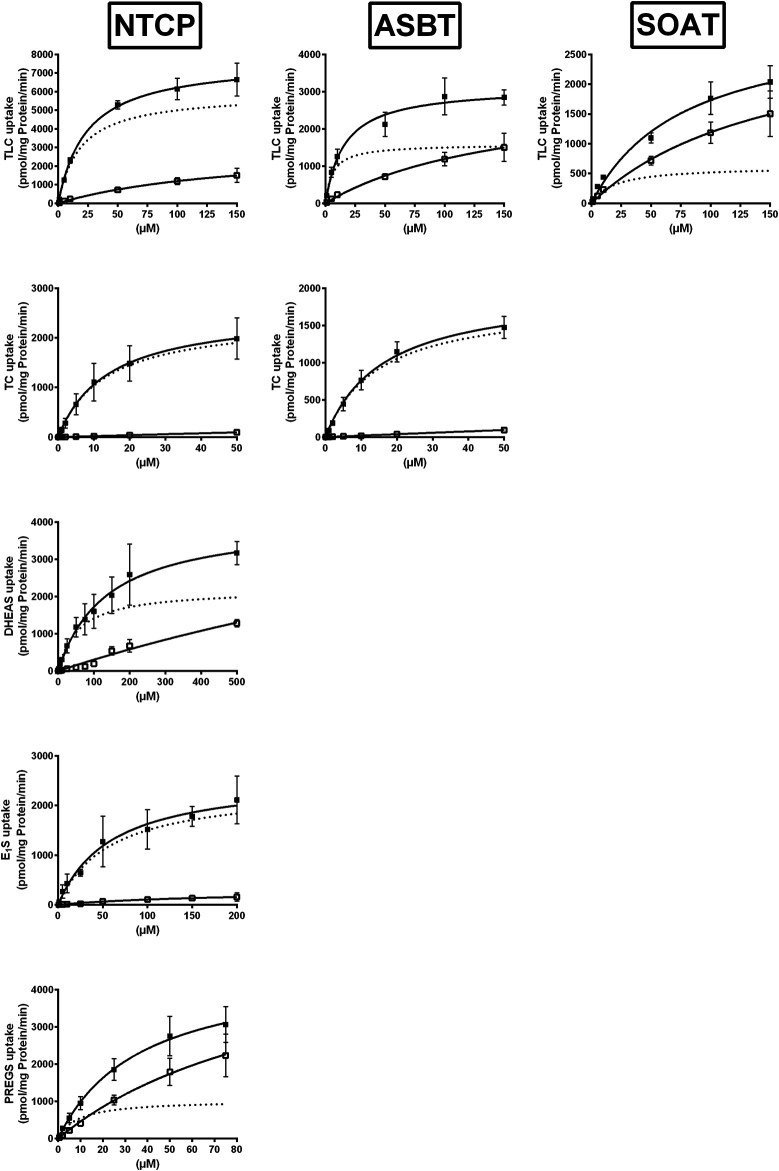
NTCP, ASBT, and SOAT transport kinetics. Concentration-dependent uptake was analyzed in NTCP-HEK293, ASBT-HEK293, and SOAT-HEK293 cells for the indicated substrates at increasing substrate concentrations. Stably transfected HEK293 cells were pretreated with 1 μg/ml tetracycline to induce carrier expression. HEK293-FlpIn cells were used as control. Uptake was analyzed for 1 min at 37°C. Afterward, the medium was removed and each cell monolayer was washed and processed to determine the protein content and cell-associated radioactivity. Specific uptake was calculated by subtracting non-specific uptake of the HEK293-FlpIn control cells (open squares) from uptake into carrier-overexpressing HEK293 cells (filled squares) and is shown by broken lines. The values represent means ± SD of duplicate experiments, each with triplicate determinations (*n* = 6). Transport kinetic parameters are summarized in Table 2.

To closer analyze the TLC transport via NTCP, ASBT, and SOAT, transport kinetics were comparatively analyzed for all three carriers. In addition, the transport kinetics for TC, DHEAS, E_1_S, and PREGS were determined. The primary transport data are presented in Figure 2 and the Michaelis–Menten parameters *K*_m_ and *V*_max_ are listed in Table 2. The apparent *K*_m_ values for TLC were within the same range for all three carriers, being 18.4, 5.9, and 19.3 µM for NTCP, ASBT, and SOAT, respectively. The *V*_max_ values ranged in the order NTCP > ASBT > SOAT. The transport kinetics for TC were well comparable between NTCP and ASBT with *K*_m_ of 13.1 and 14.7 µM as well as *V*_max_ of 2395 and 1821 pmol/mg protein/min, respectively. Also, the transport kinetics for the sulfated steroid hormones were comparable between NTCP and SOAT with *K*_m_ of 56.1 and 28.7 µM for DHEAS, as well as 8.8 and 11.3 µM for PREGS, respectively. However, SOAT showed a much lower *K*_m_ of 12.0 µM and *V*_max_ of 585 pmol/mg protein/min compared to NTCP (*K*_m_ = 57.6 µM and *V*_max_ = 2367 pmol/mg protein/min) for the substrate E_1_S. Of note, the transport data of E_1_S, DHEAS, and PREGS for SOAT were taken from a previous study, that used however exactly the same SOAT-HEK293 cell lines and measuring methodology (Geyer et al., 2007).

**TABLE 2:** Transport kinetic parameters for TLC, TC, DHEAS, E_1_S, and PREGS uptake via NTCP, ASBT, and SOAT. Michaelis-Menten kinetic parameters (*K*_m_ and *V*_max_) were calculated by nonlinear regression analysis from the primary transport data shown in Figure 2. Values represent means ± SD of combined data of two independent experiments, each with triplicate determinations (*n* = 6).

	Substrate	Apparent *K*_m_ (µM)	*V*_max_ (pmol/mg protein/min)
NTCP	TLC	18.4 ± 2.3	5915 ± 189
	TC	13.1 ± 0.8	2395 ± 59
	DHEAS	56.1 ± 8.0	2198 ± 101.7
	E_1_S	57.6 ± 11.3	2367 ± 170.8
	PREGS	8.8 ± 2.1	1036 ± 69.1
ASBT	TLC	5.9 ± 1.8	1585 ± 99.7
	TC	14.7 ± 1.5	1821 ± 75.1
SOAT	TLC	19.3 ± 6.8	617 ± 57
	*DHEAS* ^a^	*28.7* ± *3.9*	*1899* ± *81*
	*E*_*1*_ *S* ^a^	*12.0* ± *2.3*	*585* ± *34*
	*PREGS* ^a^	*11.3* ± *3.0*	*2168* ± *134*

a Values in italic taken from Geyer et al. (2007).

### TLC, TC, and DHEAS as Inhibitors of NTCP, ASBT, and SOAT

**FIGURE 3:**
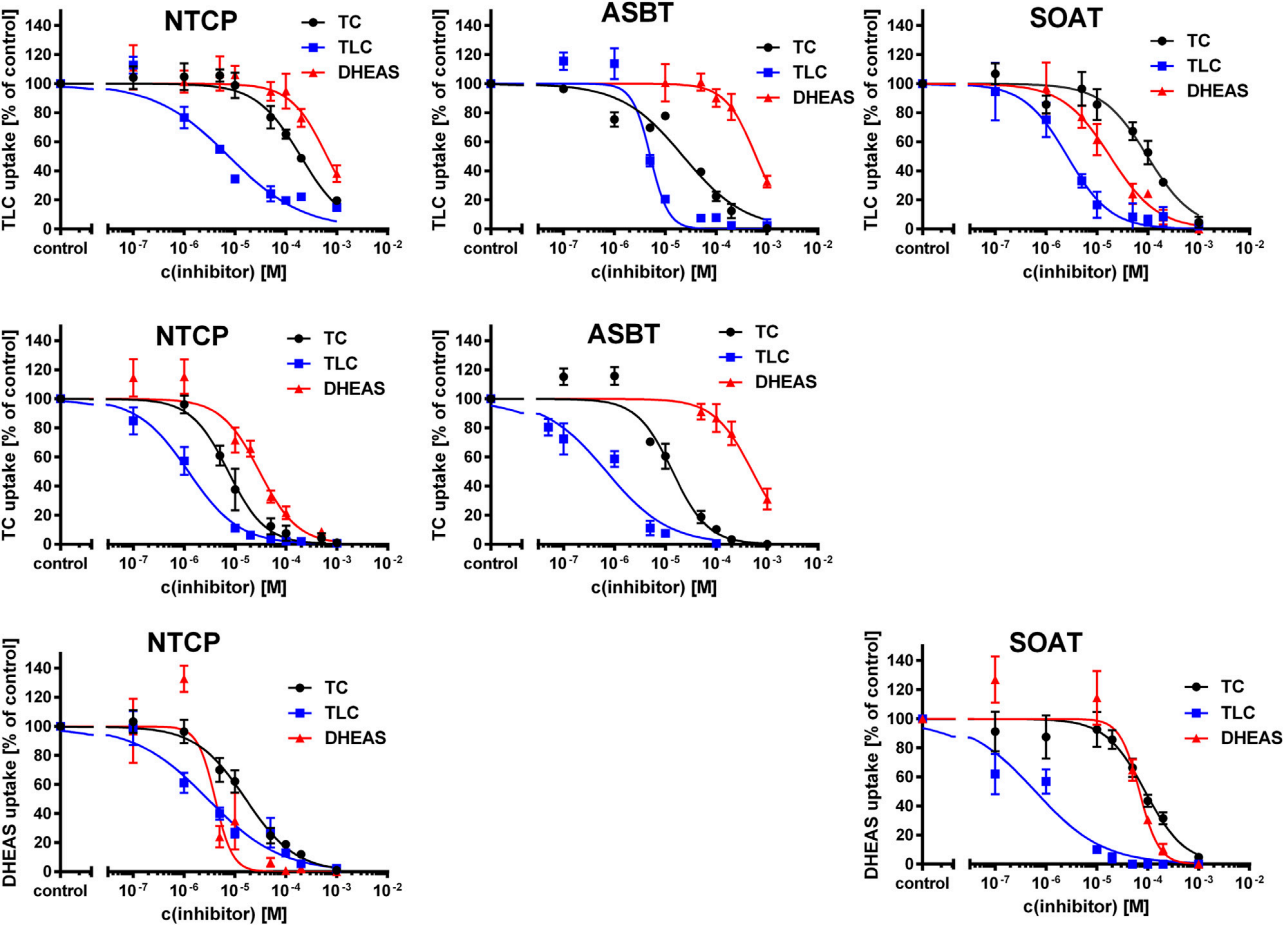
Inhibition of NTCP, ASBT, and SOAT with substrate inhibitors. Transport of [^3^H]TC, [^3^H]TLC, and [^3^H]DHEAS was analyzed in NTCP-HEK293, ASBT-HEK293, and SOAT-HEK293 cells in the presence of the substrate inhibitors TC, TLC, and DHEAS at increasing concentrations ranging from 100 nM till 1000 µM. After 5 min of preincubation with the respective inhibitory compound, 200 nM [^3^H]TLC, [^3^H]TC or [^3^H]DHEAS were added for additional 1 min and the cell lysates were analyzed by liquid scintillation counting. Unspecific uptake of HEK293-FlpIn cells was set to 0%. Carrier-specific uptake without inhibitor was set to 100%. IC_50_ values were calculated by sigmoidal dose-response (variable slope) with a goodness of fit ≥0.95 (GraphPad Prism software, version 6.05). IC_50_ values are listed in Table 3.

As shown in Figure 3, TLC, TC, and DHEAS were used at increasing inhibitor concentrations to block the transport of the respective radiolabeled transport substrates [^3^H]TLC, [^3^H]TC, and [^3^H]DHEAS at all three carriers NTCP, ASBT, and SOAT. As expected, TLC inhibited the transport of [^3^H]TLC via NTCP, ASBT, and SOAT with comparable IC_50_ values of 1.4 µM, 4.0 µM, and 2.6 µM, respectively (Table 3). Very similar was also the inhibition of the [^3^H]TC transport by increasing concentrations of TC with IC_50_ values of 5.4 µM and 5.7 µM for NTCP and ASBT, respectively. A large difference occurred, however, when DHEAS was used as inhibitor of the [^3^H]DHEAS transport via NTCP and SOAT. While an IC_50_ value of 3.4 µM was measured for NTCP, this value was much higher (at 51.7 µM) for SOAT. This is most likely a result of the stimulatory effect of DHEAS on its own transport. This was observed at low micromolar concentrations several times before (data not shown). So, DHEAS can be classified as a mixed stimulator/inhibitor. When TLC was used as inhibitor of the [^3^H]TC or [^3^H]DHEAS transport via the respective carrier, the IC_50_ values were all within the same range between 1.7 and 3.9 µM, indicating that TLC is an equipotent inhibitor of NTCP, ASBT, and SOAT, irrespective of the substrate used for transport measurements. In contrast, DHEAS showed a very different pattern, when it was used as transport inhibitor. With the transport substrate [^3^H]TLC, DHEAS was a potent inhibitor with an IC_50_ of 15 µM for SOAT, but only a weak inhibitor (IC_50_ = 431.7 µM) for NTCP, while ASBT was only inhibited by DHEAS at very high inhibitory concentrations above 1000 µM. In the same line, the IC_50_ for DHEAS inhibition of the [^3^H]TC transport was much lower for NTCP (IC_50_ = 21.5 µM) than for ASBT (IC_50_ = 453.1 µM). This indicates that ASBT is not only transport negative for DHEAS, but seems to bind DHEAS as an inhibitor only at very high concentrations. In contrast, TC although not transported by SOAT, was a moderate inhibitor of SOAT when the transport of [^3^H]TLC (IC_50_ = 143 µM) or [^3^H]DHEAS (IC_50_ = 99.7 µM) was analyzed. Overall, this indicates that TLC, TC, and DHEAS are only good inhibitors at these carriers, which are also transport positive for the respective compound. Accordingly, TC is a weak inhibitor of SOAT, and DHEAS is a very weak inhibitor at ASBT. Another interesting observation was that the transport of [^3^H]TLC can only weakly be inhibited by TC and DHEAS, even if these compounds are transported by the respective carriers. This finding may point to the presence of separate or multiple binding sites for TLC and TC/DHEAS at the respective carriers.

**TABLE 3:** Half-maximal inhibitory concentrations (IC_50_) for substrate inhibitors at NTCP, ASBT, and SOAT. [^3^H]TLC, [^3^H]TC, and [^3^H]DHEAS substrate uptake was analyzed in NTCP-HEK293, ASBT-HEK293, and SOAT-HEK293 cells in the presence of the indicated substrate inhibitors TC, TLC, and DHEAS at increasing concentrations. The primary transport data are presented in Figure 3.

Substrate	Inhibitor	NTCP (IC_50_ in µM)	ASBT (IC_50_ in µM)	SOAT (IC_50_ in µM)
[^3^H]TLC	TC	140.8	45.9	**143.0**
	TLC	1.4	4.0	2.6
	DHEAS	431.7	**>1000**	15.0
[^3^H]TC	TC	5.4	5.7	—
	TLC	1.7	1.9	—
	DHEAS	21.5	**453.1**	—
[^3^H]DHEAS	TC	14.0	—	**99.7**
	TLC	1.8	—	3.9
	DHEAS	3.4	—	51.7

Bold face: inhibitor = not transported as substrate. Underlined: Inhibitor = transported substrate.

### Betulin-Based Inhibitors of NTCP, ASBT, and SOAT

**FIGURE 4:**
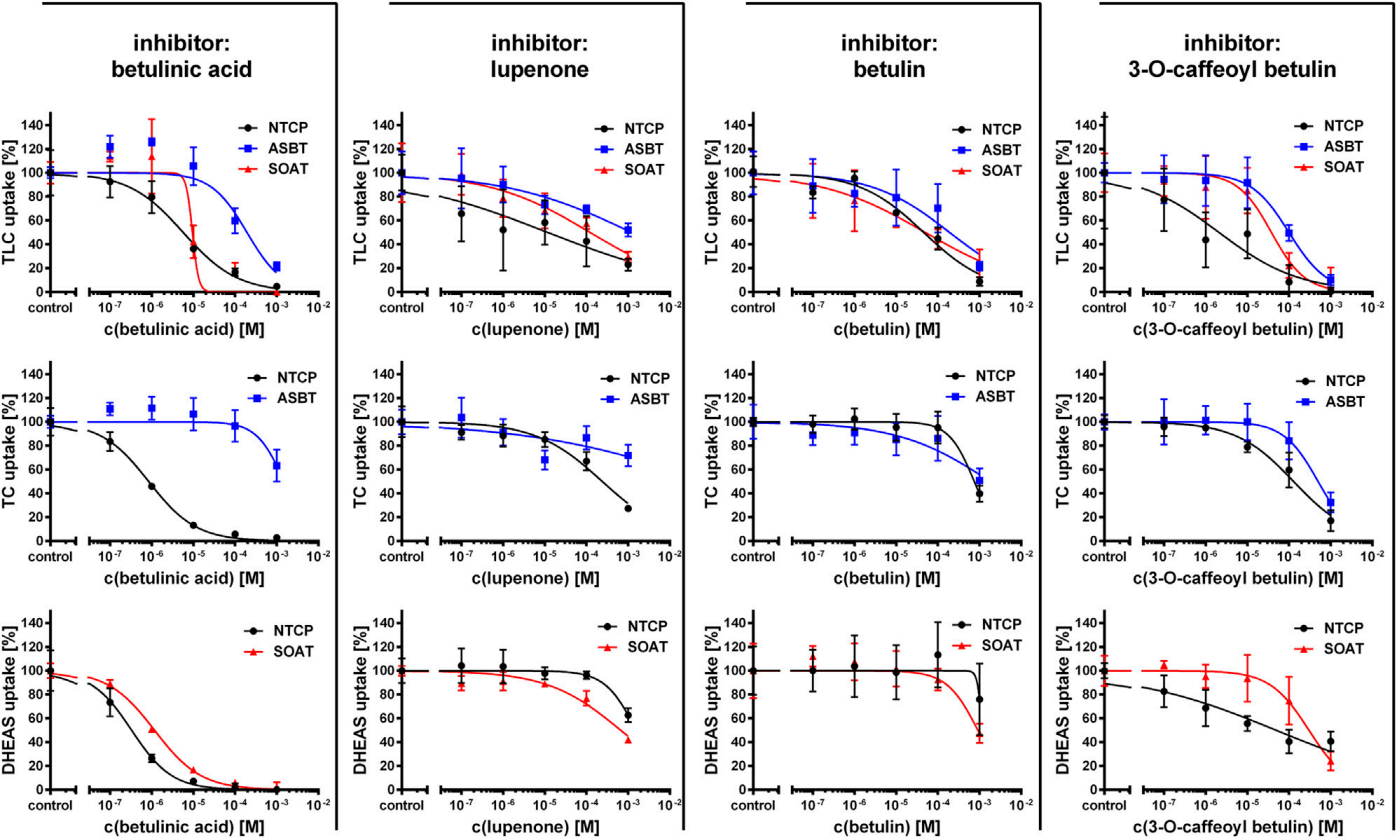
Inhibition of NTCP, ASBT, and SOAT with non-substrate betulin-based inhibitors. Transport of [^3^H]TC, [^3^H]TLC, and [^3^H]DHEAS was analyzed in NTCP-HEK293, ASBT-HEK293, and SOAT-HEK293 cells in the presence of different supposed non-substrate triterpenoid inhibitors as indicated at increasing concentrations ranging from 100 nM till 1000 µM. After 5 min of preincubation with the respective inhibitory compound, 1 µM [^3^H]TLC, [^3^H]TC or [^3^H]DHEAS were added for additional 10 min at 37°C and the cell lysates were analyzed by liquid scintillation counting. Experiments performed without inhibitor were set to 100%. Means of negative controls were subtracted to calculate net transport rates (expressed as percentage of control). Data represent means ± SD of quadruplicate determinations of representative experiments. IC_50_ values were calculated by sigmoidal dose-response (variable slope) with a goodness of fit ≥0.95 (GraphPad Prism software, version 6.05). IC_50_ values are listed in Table 4.

As the next group of inhibitors, several betulin derivatives (structures see Figure 8) were analyzed at increasing concentrations as inhibitors of the [^3^H]TLC, [^3^H]TC, and [^3^H]DHEAS transport via the respective SLC10 carriers (Table 4). As shown in Figure 4, there is no common inhibition pattern and the half-maximal inhibitory concentrations showed a correlation neither for the individual carrier, nor for the betulin derivative used as inhibitor, nor for the substrate used for the transport measurements. While some of the betulin derivatives were quite potent inhibitors for NTCP and SOAT, this was not the case for ASBT. For this carrier, the only relevant inhibition was observed for 3-O-caffeoyl betulin when [^3^H]TLC was used as substrate with an IC_50_ of 99.7 µM. In contrast, betulinic acid potently inhibited NTCP and SOAT, in particular when [^3^H]DHEAS was used as substrate (IC_50_ = 0.3 and 1.2 µM, respectively). In a similar manner, lupenone and betulin are strong inhibitors at NTCP, when [^3^H]TLC was used as the substrate, while they lost their inhibitory potency, when [^3^H]DHEAS was used as substrate. This clearly indicates that the inhibitory potency of the individual betulins not only depends on the respective carrier, but also on the substrate that is used for the transport measurements. The fact that lupenone, 3-O-caffeoyl betulin and betulin had a generally higher inhibitory potency when [^3^H]TLC was used as the substrate additionally points to separated substrate binding sites for the substrates TLC and TC/DHEAS as already emphasized above. The only exception from this rule is betulinic acid that was much more potent as inhibitor at NTCP and SOAT when [^3^H]DHEAS was used as a substrate compared to [^3^H]TLC. However, it has to be noted that it cannot be completely excluded that betulinic acid is a transported substrate of these carrier as hypothesized before (Kirstgen et al., 2020). This could explain the differing inhibition pattern of this acidic derivative compared to the other non-acidic betulin derivatives.

**TABLE 4:** Half-maximal inhibitory concentrations (IC_50_) for non-substrate triterpenoid inhibitors at NTCP, ASBT, and SOAT. [^3^H]TLC, [^3^H]TC, and [^3^H]DHEAS substrate uptake was analyzed in NTCP-HEK293, ASBT-HEK293, and SOAT-HEK293 cells in the presence of the indicated non-substrate inhibitors at increasing concentrations. The primary transport data are presented in Figure 4.

Substrate	Inhibitor	NTCP (IC_50_ in µM)	ASBT (IC_50_ in µM)	SOAT (IC_50_ in µM
[^3^H]TLC	Betulinic acid	5.7	193.3	9.5
	Lupenone	11.8	>1000	122.4
	3-O-Caffeoyl betulin	2.1	99.7	37.8
	Betulin	45.8	191.5	50.1
[^3^H]TC	Betulinic acid	0.8	>1000	—
	Lupenone	240.4	>1000	—
	3-O-Caffeoyl betulin	128.7	498.6	—
	Betulin	747.9	>1000	—
[^3^H]DHEAS	Betulinic acid	0.3	—	1.2
	Lupenone	>1000	—	664.5
	3-O-Caffeoyl betulin	49.1	—	301.1
	Betulin	>1000	—	912.2

### Non-Steroidal Inhibitors of NTCP, ASBT, and SOAT

The inhibitor screening was also extended to compounds that previously were identified as non-steroidal inhibitors of NTCP or SOAT, including cyclosporine A, ezetimibe, bromosulfophthalein (BSP), irbesartan, losartan, troglitazone, erythrosine B, and ginkgolic acid 17:1 (Table 5, selected structures see Figure 8). All these compounds not only inhibited the transport function of NTCP, but also its role as HBV/HDV entry receptor in previous studies (Fukano et al., 2019). Therefore, these experiments also aimed to analyze the cross-reactivity of these antiviral drug candidates at the most NTCP-related proteins ASBT and SOAT. For these measurements, again all three potential transport substrates, [^3^H]TLC, [^3^H]TC, and [^3^H]DHEAS, were analyzed in the absence (positive control) and the presence of a fixed 100 µM inhibitor concentration. All inhibitors then were graded as strong, medium or weak inhibitor based on the residual transport activity of 0–19% (+++), 20–49% (++) and 50–79% (+), respectively, in the presence of inhibitor. Generally, these NTCP inhibitors showed significant cross-reactivity with SOAT and/or ASBT, at least with one of the investigated transport substrates. Therefore, compounds that were previously classified as specific NTCP inhibitors should rather be considered pan-SLC10 inhibitors. One exception was ezetimibe that only showed weak inhibitory potential at NTCP exclusively for TC as substrate. As previously shown for the betulin derivatives, the choice of the investigated SLC10 carrier substrate can affect the classification as inhibitor. As an example, losartan and ginkgolic acid 17:1 showed identical inhibitory classification for TLC and TC at NTCP, as well as for TLC and DHEAS at SOAT. However, their inhibitory potential for DHEAS as the transported substrate at NTCP revealed huge difference in classification. In contrast, irbesartan and cyclosporine A showed similar inhibition pattern at NTCP and SOAT, irrespective of the substrate used for transport measurements. However, irbesartan and cyclosporine A did not inhibit ASBT at all. This indicates that the transport of a particular substrate ([^3^H]TLC, [^3^H]TC, [^3^H]DHEAS) can be differentially addressed with a particular inhibitor, what would support the idea of different substrate binding sites in the SLC10 carriers.

**TABLE 5:** Inhibitor screening for NTCP, ASBT and SOAT. [^3^H]TLC, [^3^H]TC, and [^3^H]DHEAS substrate uptake was analyzed in NTCP-HEK293, ASBT-HEK293, and SOAT-HEK293 cells in the presence of the indicated inhibitors at 100 µM inhibitory concentrations. Grading of the net uptake compared to the non-inhibited control was: “−” for 80–100% residual uptake of the respective substrate compared to positive control (w/o inhibitor), “+” for 50–79% residual uptake (weak inhibition), “++” for 20–49% residual uptake (medium inhibition) and “+++” for 0–19% residual uptake (strong inhibition). Experiments were performed in duplicate each with triplicate determinations (*n* = 6).

Inhibitor	NTCP substrate			ASBT substrate		SOAT substrate	
	TLC	TC	DHEAS	TLC	TC	TLC	DHEAS
Cyclosporine A	+	++	+++	−	−	+	+
Ezetimibe	−	+	−	−	−	−	−
BSP	+	++	++	+	++	+++	+++
Irbesartan	+	++	+++	−	−	+++	++
Troglitazone	+++	+++	++	+++	+++	+++	+++
Erythrosine B	+	+++	+	+	+	+	++
Ginkgolic acid 17:1	+	+	++	−	−	++	+++
Losartan	+	+	−	−	−	++	+++

### Cross-Inhibition of the HBV/HDV-Derived Myr-preS1 Peptide with TLC, TC, and DHEAS

**FIGURE 5:**
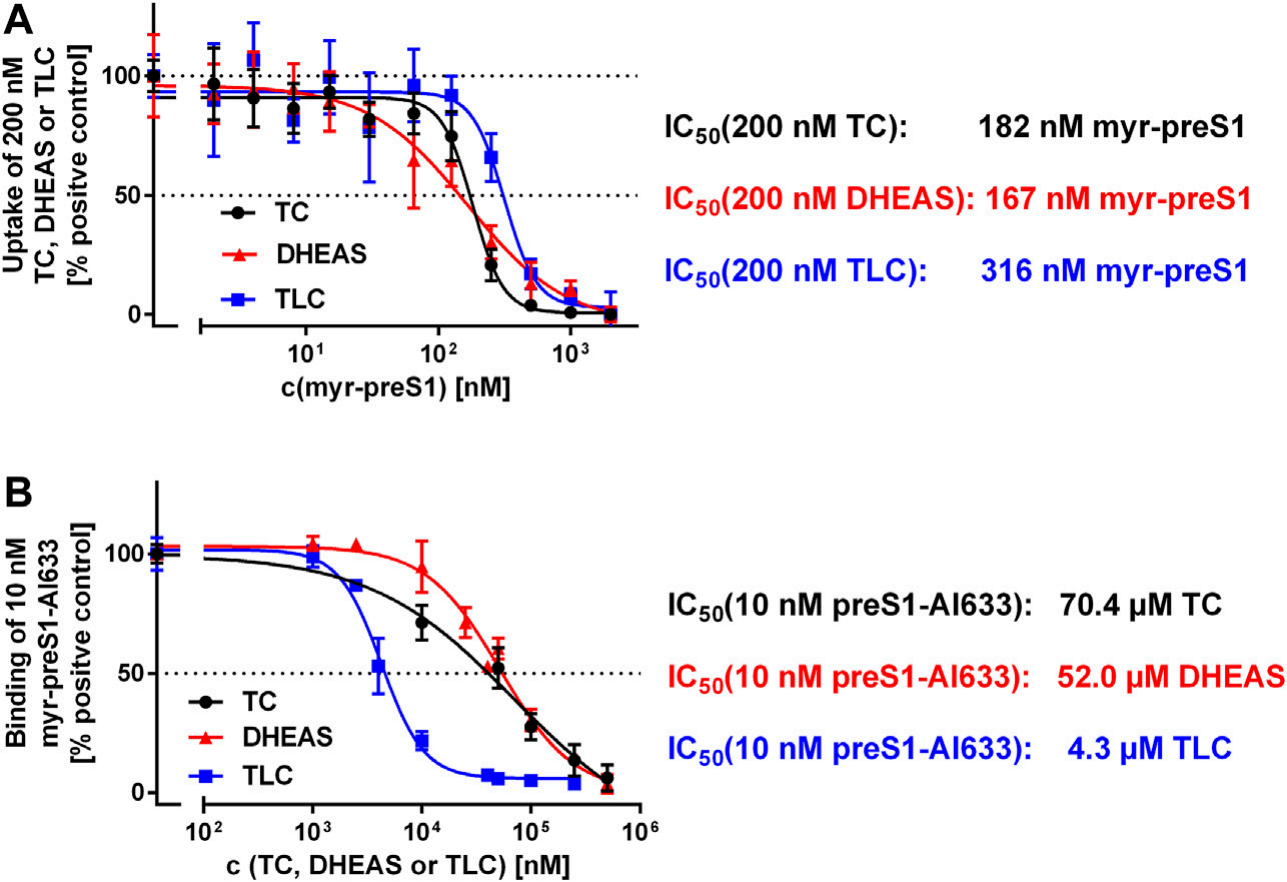
Binding **(A)** and inhibition **(B)** of the myr-preS1 peptide at NTCP-HEK293 cells. **(A)** Transport of [^3^H]TC, [^3^H]DHEAS, and [^3^H]TLC was analyzed in NTCP-HEK293 cells in the presence of increasing concentrations of the myr-preS1 peptide. Transport rates without myr-preS1 peptide were set to 100%. Graphs show combined data of at least two independent experiments and represent means ± SD. **(B)** Binding of the fluorescent myr-preS1-Al633 peptide was analyzed in NTCP-HEK293 cells in the presence of increasing concentrations of TC, DHEAS, or TLC. Graphs show combined data of at least two independent experiments and represent means ± SD. Fluorescence was quantified by a fluorescence reader and values are expressed as percentage of control. IC_50_ values were calculated by sigmoidal dose-response (variable slope) with a goodness of fit ≥0.95 (GraphPad Prism software, version 6.05).

We used two different assays to analyze the myr-preS1 peptide binding behavior in dependence of the substrate used. First, [^3^H]TC, [^3^H]TLC, and [^3^H]DHEAS transport was analyzed at increasing concentrations of myr-preS1 serving as inhibitor of transport (Figure 5A). Interestingly, myr-preS1 was quasi equipotent for the inhibition of the [^3^H]TC and [^3^H]DHEAS transport with IC_50_ values of 182 nM and 167 nM, respectively. In contrast, higher concentrations were needed for half-maximal inhibition of the [^3^H]TLC transport (IC_50_ = 316 nM). In a second assay, the binding of the myr-preS1 peptide to NTCP was analyzed in the presence of increasing concentrations of TLC, TC, and DHEAS serving as peptide binding inhibitors (Figure 5B). Again, TC and DHEAS were quasi equipotent in this assay with IC_50_ values of 70.4 µM and 52.0 µM, respectively, whereas an order of magnitude lower concentrations of TLC were needed to replace the myr-preS1 peptide from its binding sites at NTCP (IC_50_ = 4.3 µM). This again underlines the different behavior of TLC in its binding to NTCP compared with TC and DHEAS.

### Comparative Analysis of NTCP, ASBT, and SOAT Homology Models

**FIGURE 6:**
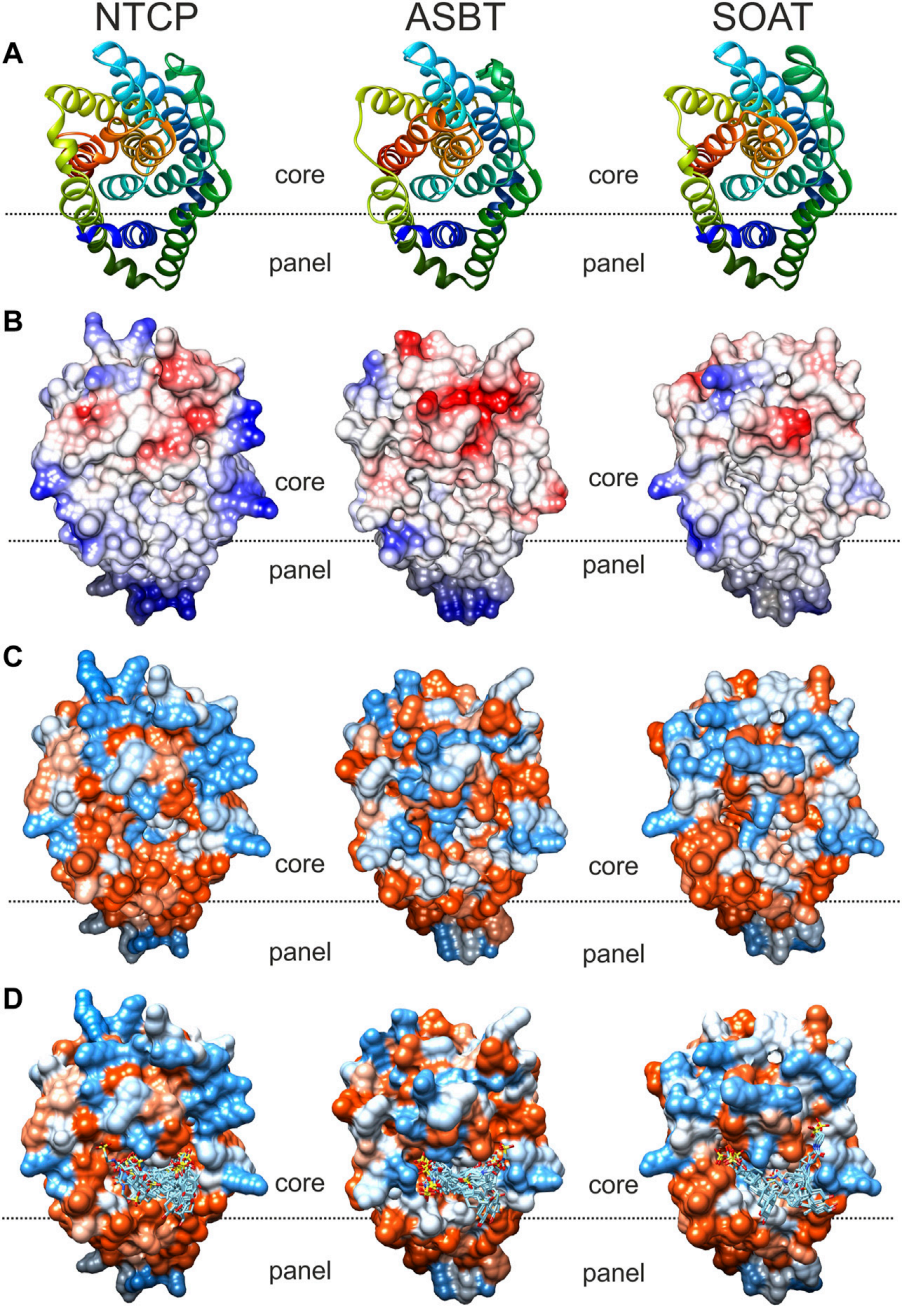
Comparative substrate docking to homology models of NTCP, ASBT, and SOAT in outward-open conformation. Based on the crystal structure of bacterial Asbt_Yf_ (4n7x.1.a) outward open homology models of the SLC10 transporters NTCP, ASBT and SOAT were generated with SWISS-MODEL. All models are shown in identical orientation in top view. The dotted line gives an orientation for the localization of the proposed substrate-binding cavity between the panel and core domains of the respective carrier. **(A)** Backbone structure colored in rainbow-mode to depict similar transmembrane structure. **(B)** Molecular surface colored due to Coulomb force calculation (electrostatic forces); starting from −10 (red), over 0 (white), up to +10 (blue). **(C)** Hydrophobicity surface coloring due to amino acid residue sequence hydrophobicity ranging from hydrophilic (blue) over neutral (white) to lipophilic (red). **(D)** *In silico* docking of TLC by SwissDock. Docked clusters were reduced to TLC molecules in reasonable proximity to the putative outward facing binding pocket. All models were visualized with the UCSF CHIMERA software.

In order to visualize the 3-dimensional structures of NTCP, ASBT and SOAT, homology models were generated as shown in Figure 6. The backbone structures (Figure 6A) of the three carriers are very similar and are composed of nine transmembrane domains (TMDs). Structurally, the proteins can be subdivided into a core domain, composed of TMDs 2-4 and 7-9, and a panel domain, composed of TMDs 1, 5 and 6, according to the structures of the bacterial proteins Asbt_Nm_ (PDB: 3ZUY.A) and Abst_Yf_ (PDB: 4N7X.A) (Hu et al., 2011; Zhou et al., 2014). For homology modeling, the outward-open Asbt_Yf_ model (PDB: 4N7X.1.A) was used that shows an outward-exposed substrate binding cavity between the panel and core domains as recently verified (Wang et al., 2021). The molecular surface of the NTCP, ASBT and SOAT homology models was colored according to Coulomb potential (Figure 6B) or by amino acid residue hydrophobicity (Figure 6C) and revealed significant differences for both physical parameters. Therefore, no common pattern can be recognized in the substrate binding cavities of NTCP, ASBT, and SOAT. In addition, the models were used for *in silico* docking with TLC as the ligand, using the docking module of SWISS-Dock. As shown in Figure 6D, TLC showed several potential docking/binding sites at the proposed substrate binding cavities and in this case showed partly overlapping orientation for the three carriers NTCP, ASBT, and SOAT (Figure 6D).

### Comparative Analysis of NTCP, ASBT, and SOAT Substrate Pharmacophore Models

**FIGURE 7:**
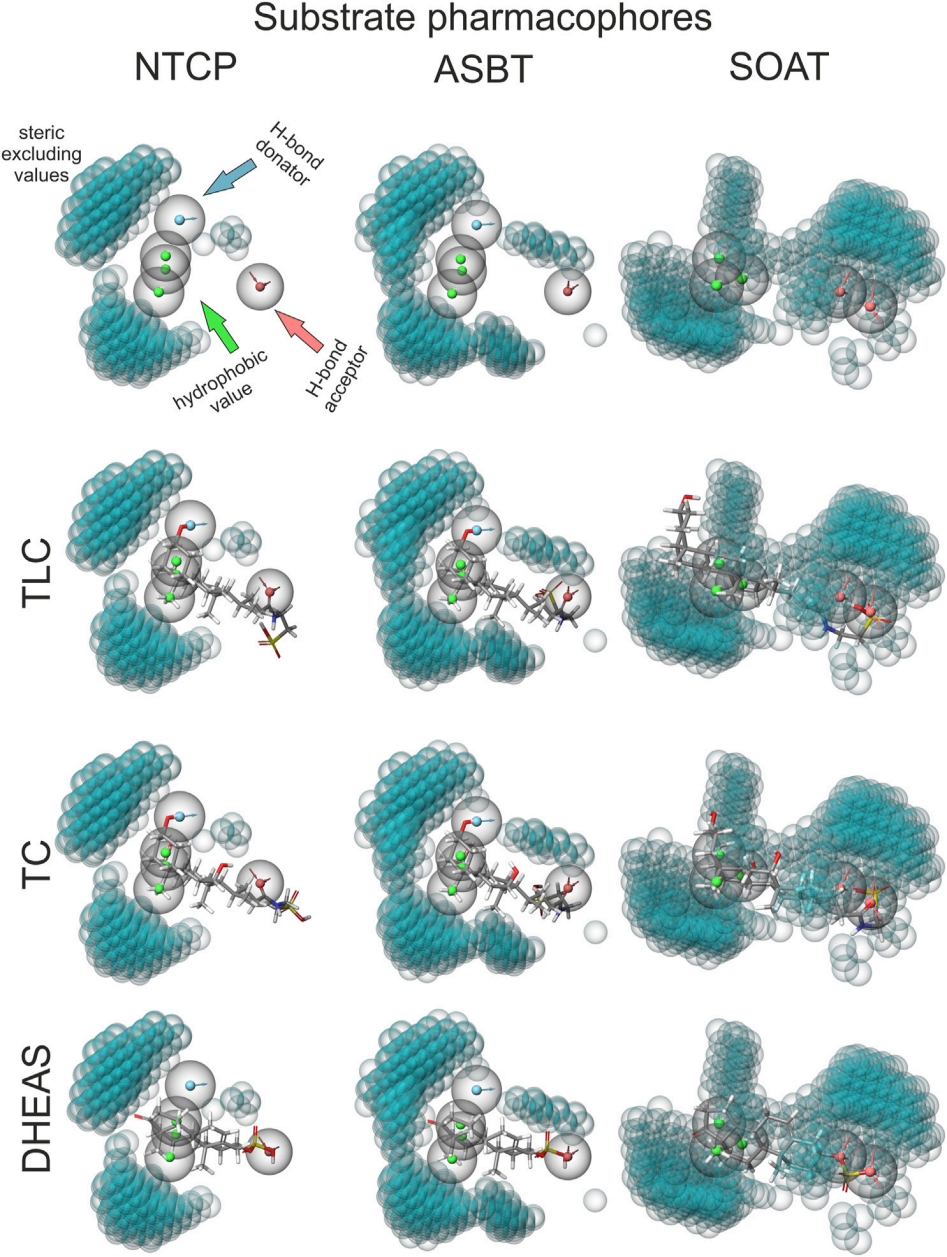
TLC, TC and DHEAS fitted to substrate pharmacophore models for NTCP, ASBT, and SOAT. Pharmacophore models were generated via the MAESTRO Molecular Modeling Interface (Version 12.2) of SCHRÖDINGER software with all substrates listed in Table 1. For size comparison: steric excluded volume sphere radii 1 Å (little blue spheres). H-bond donators are depicted as spheres with a blue core and outward facing arrows. H-bond acceptors are depicted as spheres with a red core and inward facing arrows. Hydrophobic values are depicted as spheres with green cores. Top line shows the calculated empty pharmacophores. Lines below show best fitting of TLC, TC and DHEAS into the respective pharmacophores.

**FIGURE 8:**
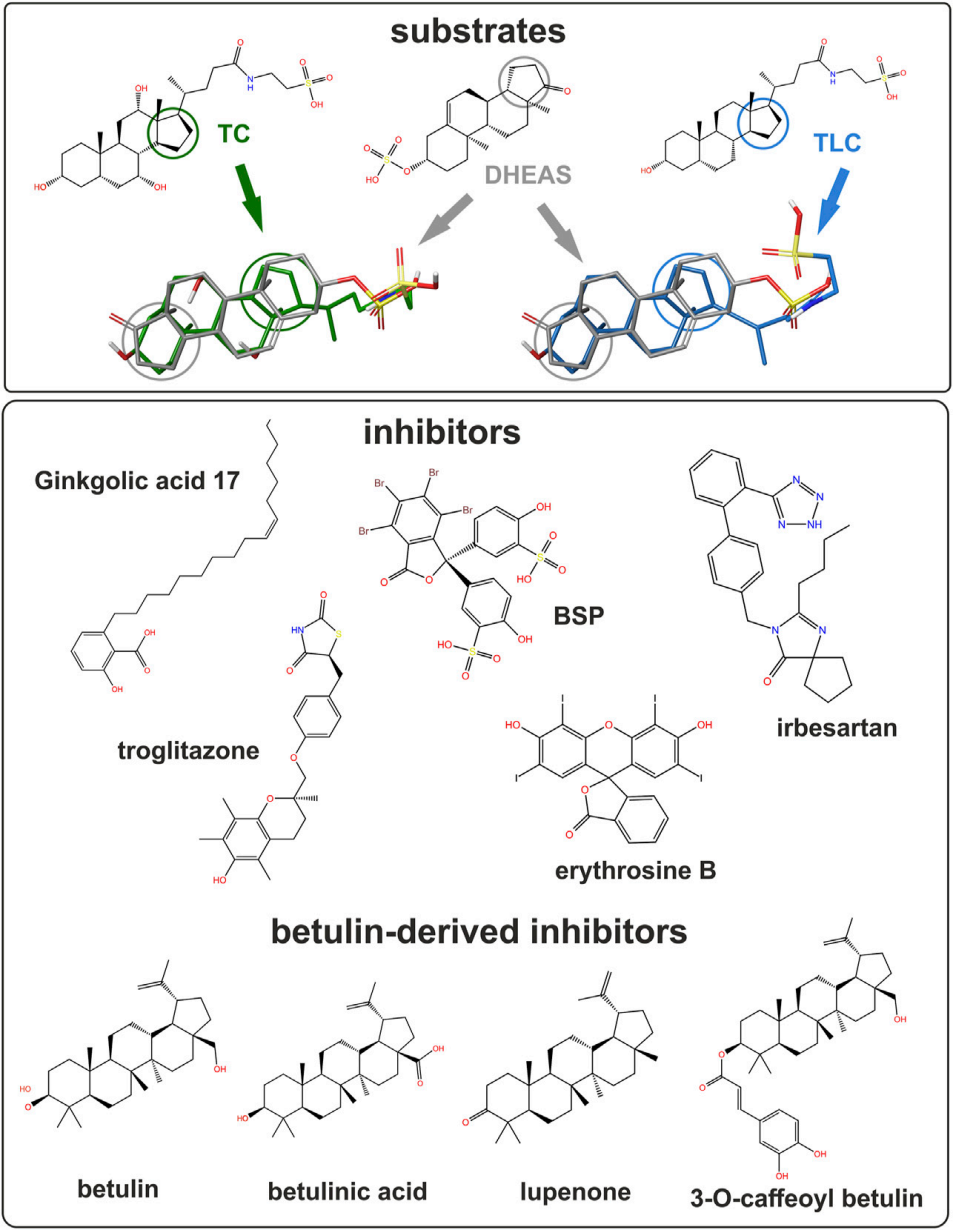
Structure of studied substrates and inhibitors. Selected compounds shown for structural comparison as 2D structure formula (PubChem ID - CID). Substrates: taurocholic acid - TC (CID 6675), taurolithocholic acid- TLC (CID 439763) and DHEAS (CID 12594). Inhibitors: ginkgolic acid 17:1 (CID 5469634), BSP (CID 5345), irbesartan (CID 3749), troglitazone (CID 5591) and erythrosine B (CID 3259). Betulin-derived inhibitors: betulin (CID 72326), betulinic acid (CID 64971), lupenone (CID 92158) and 3-O-caffeoyl betulin (CID 10153267). 3D overlays illustrate DHEAS (grey), TC (green), or TLC (blue). Colored circles indicate the orientation of the steroid backbone.

Finally, the substrate recognition pattern of NTCP, ASBT, and SOAT was visualized by common substrate pharmacophore modeling based on the data shown in Table 1. The pharmacophores are presented for each transporter in the first line of Figure 7. In addition, the SLC10 substrates TLC, TC and DHEAS are fitted into all pharmacophore models. The NTCP and ASBT pharmacophores are quite similar and are characterized by one hydrogen bond donator, one hydrogen bond acceptor and three hydrophobic features that all are similarly oriented to each other. However, in comparison to NTCP, ASBT revealed much more excluding values. As consequence, DHEAS does not fit into the ASBT pharmacophore model due to steric overlap of the 3′ sulfate group with the excluded space. In contrast, TC and TLC fit quite well in both pharmacophores of NTCP and ASBT. The pharmacophore model of SOAT is significantly different from those of NTCP and ASBT and revealed two hydrogen bond acceptor groups and three hydrophobic features. In addition, the SOAT pharmacophore is significantly restricted by spacious excluding values. As consequence, TC does not fit into this pharmacophore model due to steric overlap of the 7′ and 12′ hydroxyl groups with the excluded space. In contrast, the flat DHEAS molecule fits perfectly into this pharmacophore. Interestingly, also TLC fits into the SOAT pharmacophore. While the terminal sulfate group of the taurine residue covers one of the hydrogen bond acceptor groups that is occupied by the 3′ sulfate group in the case of DHEAS, the bent steroid rings A and B stretch out into the free space of the pharmacophore. This suggests substrate recognition of DHEAS and TLC in an antiparallel manner (see Figure 8). However, this is only possible when the BA molecule is not additionally hydroxylated, as it is the case for TC.

## Discussion

### Physiological Relevance of NTCP, ASBT, and SOAT

The present study suggests overlapping substrate and inhibitor binding sites for the SLC10 carriers NTCP, ASBT, and SOAT. Nevertheless, each carrier has a unique substrate spectrum to fulfill its tissue-specific role for the cellular uptake of BAs and/or sulfated steroid hormones. Based on the current knowledge, BA transport via NTCP in the liver and via ASBT in the ileum is essential for the maintenance of the enterohepatic circulation of BAs (Geyer et al., 2006) and so the physiological role of both carriers in BA transport is quite clear. In addition, NTCP could be important for the hepatic excretion of sulfated steroid hormones, such as DHEAS (Geyer et al., 2017). In contrast, a transport function for sulfated steroids would not make sense physiologically for ASBT, because relevant levels of sulfated steroid hormones are not present in the lumen of the ileum. SOAT is more widespread in its expression and was detected in germ cells of the testis, skin, placenta, mammary gland and some other hormone-dependent tissues (Geyer et al., 2007; Fietz et al., 2013; Grosser et al., 2013; Karakus et al., 2018). By SOAT-mediated uptake of sulfated steroid hormones from the blood and subsequent cleavage by the steroid sulfatase (so-called intracrine steroid synthesis) seems to contribute to the steroid regulation of many hormone-dependent tissues (Geyer et al., 2017). Apart from steroid hormones, BAs gained increasing attention as signaling molecules with broad metabolic effects acting via membrane bound (TGR5) and nuclear (FXR) receptors (Di Ciaula et al., 2017). Against this background, the transport of TLC via SOAT could also be of physiological relevance in the periphery. TLC is formed in the liver by taurine conjugation of lithocholic acid that is absorbed from the gut as secondary BA independent from carrier-/ASBT-mediated uptake. However, TLC excreted into the duodenum via bile can also be reabsorbed via ASBT in the terminal ileum before this compound undergoes bacterial de-conjugation in more distal parts of the gut. TLC then can be taken up from the portal blood into hepatocytes by NTCP. In addition to this intestinal and hepatic transport of TLC, SOAT might play a role for TLC transport in peripheral organs. Of note, TLC is getting increasing attention as signaling and regulatory molecule and SOAT could be involved in its distribution. As an example, TLC recently showed to induce relaxation of human and mouse peripheral airways that were pre-contracted by acetylcholine stimulation (Urso et al., 2020). As SOAT is also expressed in the lung (Fietz et al., 2013), SOAT-mediated uptake of TLC might be of relevance there.

### Phylogenetic Relationship and Substrate Recognition of NTCP, ASBT, and SOAT

Based on Bayesian phylogenetic analysis we previously reported that the genes coding for ASBT (*SLC10A2*) and SOAT (*SLC10A6*) emerged from a common ancestor by gene duplication (Geyer et al., 2006). This finding is supported by the very close homology of the gene structure of both genes with six coding exons and highly conserved sequences at the exon-intron boundaries. In the same way, the genes for NTCP (*SLC10A1*) and *SLC10A4* emerged from a common ancestor gene, but both genes are less homologous as indicated by the number of five (*SLC10A1*) and three (*SLC10A4*) coding exons. Even earlier, both subclades (*SLC10A2*/*SLC10A6* and *SLC10A1*/*SLC10A4*) have a common ancestor (see Supplementary Figure S1). Based on this, it is surprising that ASBT and SOAT show a contrary substrate spectrum. While ASBT is specific for BA transport, SOAT is a specific transporter for sulfate steroid hormones. NTCP, in contrast, has a much wider substrate spectrum and can transport both substrate groups and some additional compounds such as estradiol-3β**-**D-glucuronide that are not transported by ASBT and SOAT (Figure 1). The most likely explanation for this functional divergence is that the common ancestor of NTCP, ASBT and SOAT already incorporated all functions. While these functions for BA and steroid sulfate transport then were split into ASBT and SOAT, respectively, NTCP maintained the full substrate spectrum of the common ancestor. This hypothesis is supported by the fact that SOAT can still bind BAs even if they are not transported and the binding sites for BAs and steroid sulfates even seem to overlap. Accordingly, the transport of [^3^H]DHEAS via SOAT can be potently inhibited with TC (Figure 3) and several other BAs (Geyer et al., 2007). However, this does not fully apply for ASBT, which only can be inhibited with DHEAS at very high inhibitor concentrations. Therefore, ASBT seems to have lost the binding and transport function for sulfated steroids during emergence from the common SOAT/ASBT ancestor. For NTCP, an overlapping substrate-binding site for BAs (TC) and sulfated steroids (DHEAS) can be proposed. Both compounds are transported by NTCP and both are quite potent in inhibiting the transport of the respective other compound. The IC_50_ for inhibition of the [^3^H]TC transport via NTCP by DHEAS was determined to 21.5 µM, while inhibition of the [^3^H]DHEAS transport via NTCP by TC was at 14.0 µM and so within the same range. However, it has to be mentioned that the human NTCP*2 polymorphism, characterized by the amino acid substitution S267F, showed reduced transport activity for TC, but not for E_1_S, pointing to structural differences in the substrate recognition of these two substrates (Ruggiero et al., 2021).

### NTCP, ASBT, and SOAT Share Different Overlapping Substrate/Inhibitor Binding Sites

Until now, it was considered that the substrate spectra of ASBT and SOAT have no overlap. Yet in the present study, we demonstrate that TLC can be transported by all three sodium-dependent SLC10 carriers, namely NTCP, ASBT and SOAT. Therefore, the present study established TLC as the first common substrate of these carriers. However, the question if TLC was also a substrate of the common NTCP/ASBT/SOAT ancestor or if all three carriers later acquired the TLC transport function cannot be finally answered. However, it seems likely that TLC is not only a common, but also an ancient substrate of the SLC10 carriers. Anyhow, the highly conserved transport activity of all three carriers for TLC raises some questions about the exact mode of substrate recognition of this unique substrate.

Substrate recognition of TLC might occur by chance in the substrate-binding site of DHEAS, as TLC shows some structural overlap with DHEAS when oriented in an antiparallel manner, as illustrated in Figure 8. In this scenario, the terminal sulfate group of TLC would be recognized instead of the 3′ sulfate group of DHEAS, while extensive overlapping hydrophobic interactions are possible via the unmodified steroid core structures of both molecules. Indeed, DHEAS and TLC showed an antiparallel fitting into the SOAT pharmacophore model as shown in Figure 7. This is, however, not possible when TLC is additionally hydroxylated at the 7′ and/or 12’ positions, as it is the case for the BAs TCA, TCDCA, or TDCA, which all are not transported by SOAT. According to this hypothesis, TLC would be recognized by the DHEAS substrate-binding site of SOAT, while it binds as a substrate to the BA binding site in NTCP and ASBT.

A second explanation could be a separate substrate-binding site for TLC that is conserved in all three carriers NTCP, ASBT and SOAT. Although this proposed TLC binding site seems to partly overlap with the binding sites for TC and DHEAS, it would allow TLC transport independent from the TC/DHEAS transport activity of the respective carrier. Several findings of the present study support this hypothesis: (I) TLC was found to be an equipotent inhibitor of all three carriers NTCP, ASBT, and SOAT irrespective of the substrate that was used to measure transport activity. This finding can be explained by common binding and transport of TLC for all three carriers and thereby unspecific inhibition of the transport of any other substrate. (II) As the transport of [^3^H]TLC can only weakly be inhibited by TC and DHEAS, even if these compounds are transported by the respective carriers, TLC transport seems to occur independent from TC/DHEAS binding and transport. (III) The non-substrate inhibitors lupenone, 3-O-caffeoyl betulin and betulin had much higher inhibitory potency when [^3^H]TLC was used as the transport substrate compared with [^3^H]TC and [^3^H]DHEAS, indicating that the inhibitor binding site of these betulin derivatives closer overlaps with the TLC binding site compared with the TC/DHEAS binding site.

A broader interpretation of this hypothesis would propose a larger substrate/inhibitor entry zone in the outward oriented space between the core and the panel domain that is characterized by multiple interaction domains for the different substrates (TLC, TC and DHEAS) and inhibitors. This would explain the large cross-inhibition pattern between the different substrate and inhibitor groups. From this entry zone, single or multiple pathways for substrate transport through the transporter protein might exist or only binding of a transport substrate might indeed induce conformational changes that open the substrate-binding zone to the intracellular compartment for substrate release. This scenario would also explain the existence of pan-SLC10 substrates (TLC) and inhibitors (e.g. erythrosine B, troglitazone, or BSP), while other substrates (TC, DHEAS) and inhibitors (e.g. irbesartan, cyclosporine A, ginkgolic acid 17:1) are specific for a subgroup of SLC10 carriers.

Interestingly, the myr-preS1 lipopeptide showed equipotent inhibition of all substrates (TC, TLC, and DHEAS) of NTCP, suggesting that this peptide completely blocks the access of any substrate to its respective binding site. However, TLC was much more potent in blocking the binding of the myr-preS1 peptide from NTCP compared with TC and DHEAS. This could also be explained by the particular *trans*-inhibitory potential of this compound at NTCP (Lowjaga et al., 2021). TLC, after carrier-mediated uptake or passive diffusion, can bind to an intracellular TLC binding site of NTCP and thereby *trans*-inhibits myr-preS1 peptide and HDV binding from the outside of the cell. Interestingly, this *trans*-inhibitory effect of TLC also inhibited *in vitro* HDV infection of NTCP expressing HepG2 hepatoma cells (Lowjaga et al., 2021). In contrast, such a *trans*-inhibitory effect is not known for the substrates TC and DHEAS, what could explain their lower potential to inhibit myr-preS1 binding to NTCP.

The existence of overlapping multiple substrate binding sites as proposed in the present study for NTCP, ASBT, and SOAT, was described for several other carrier proteins before. As an example, mutagenesis studies of the rat Organic cation transporter 1 (rOct1) revealed overlapping binding sites for different substrates and allosteric effectors (Koepsell, 2019). The multidrug efflux transporter MDR1 P-glycoprotein (syn. ABCB1) is another example of a carrier with multiple substrate binding sites. P-glycoprotein seems to have a large drug-binding pocket with different overlapping sites for binding of individual substrate groups. Thereby, P-glycoprotein can recognize and transport a vast variety of structurally unrelated drugs and toxins (Chufan et al., 2015).

### Cross-Reactivity of Pharmacological Inhibitors of SLC10 Carriers and Clinical Implications

The discovery of NTCP as a high-affinity receptor for HBV and HDV opened the field for the development of HBV/HDV entry inhibitors, preferably based on small molecules with oral bioavailability (Yan et al., 2012; König et al., 2014). In a previous study, we could demonstrate that small molecules from the group of pentacyclic triterpenoids, including betulinic acid and lupenone, show anti-HDV activity *in vitro* making them attractive virus entry inhibitor candidates (Kirstgen et al., 2020). However, as demonstrated in the present study, both compounds show significant cross-reactivity with SOAT, while ASBT transport was not affected by these betulin derivatives. This exemplifies that inhibitors of NTCP, ASBT, and SOAT should principally tested for cross-reactivity against the other SLC10 carriers. In conclusion, NTCP, ASBT, and SOAT are interesting drug targets and several pharmacological inhibitors have already been established against these carriers. In the present study, overlapping substrate and inhibitor binding sites are proposed that are differently active in NTCP, ASBT, and SOAT. TLC was identified as the first common substrate for all three carriers and it was clearly shown that most of the SLC10 inhibitors are not carrier-specific, but rather cross-react at least with one of the other related SLC10 carriers. This should be considered when pharmacological inhibitors are developed against NTCP, ASBT, or SOAT.

## Data Availability

The original contributions presented in the study are included in the article/Supplementary Material, further inquiries can be directed to the corresponding author.

## Glossary

CA: Cholic acid
CDCA: Chenodeoxycholic acid
DCA: Deoxycholic acid
UDCA: Ursodeoxycholic acid
LCA: Lithocholic acid
Sarcosine CA: Sarcosine cholic acid
GCA: Glycocholic acid
GDCA: Glycodeoxycholic acid
GCDCA: Glycochenodeoxycholic acid
GUDCA: Glycoursodeoxycholic acid
TCA/TC: Taurocholic acid
TDCA: Taurodeoxycholic acid
TCDCA: Taurochenodeoxycholic acid
TUDCA: Tauroursodeoxycholic acid
TLCA/TLC: Taurolithocholic acid
DHEAS: Dehydroepiandrosterone sulfate
PREGS: Pregnenolone sulfate
E_1_S: Estrone-3-sulfate
E-3β-D-G: Estrone-3β-D-glucuronide
E-17β-D-G: Estradiol-17β-D-glucuronide
HBV: Human Hepatitis B Virus
HDV: Human Hepatitis D Virus
NTCP: Na^+^/taurocholate cotransporting polypeptide (SLC10A1)
ASBT: Apical sodium-dependent bile acid transporter (SLC10A2)
SOAT: Sodium-dependent organic anion transporter (SLC10A6)

## References

[B1] Al-Dury S. Marschall H.-U. (2018). Ileal Bile Acid Transporter Inhibition for the Treatment of Chronic Constipation, Cholestatic Pruritus, and NASH. Front. Pharmacol. 9, 931. 10.3389/fphar.2018.00931 30186169 PMC6111463

[B2] Ananthanarayanan M. Ng O. C. Boyer J. L. Suchy F. J. (1994). Characterization of Cloned Rat Liver Na(+)-Bile Acid Cotransporter Using Peptide and Fusion Protein Antibodies. Am. J. Physiol.-Gastrointestinal. Liver Physiol. 267 (4 Pt 1), G637–G643. 10.1152/ajpgi.1994.267.4.G637 7943329

[B3] Chufan E. E. Sim H.-M. Ambudkar S. V. (2015). Molecular Basis of the Polyspecificity of P-Glycoprotein (ABCB1). Adv. Cancer Res. 125, 71–96. 10.1016/bs.acr.2014.10.003 25640267 PMC7709800

[B4] Claro da Silva T. Polli J. E. Swaan P. W. (2013). The Solute Carrier Family 10 (SLC10): beyond Bile Acid Transport. Mol. Aspects Med. 34 (2, 3), 252–269. 10.1016/j.mam.2012.07.004 23506869 PMC3602841

[B5] Craddock A. L. Love M. W. Daniel R. W. Kirby L. C. Walters H. C. Wong M. H. (1998). Expression and Transport Properties of the Human Ileal and Renal Sodium-dependent Bile Acid Transporter. Am. J. Physiol.-Gastrointestinal Liver Physiol. 274 (1), G157–G169. 10.1152/ajpgi.1998.274.1.G157 9458785

[B6] Di Ciaula A. Garruti G. Lunardi Baccetto R. Molina-Molina E. Bonfrate L. Wang D. Q.-H. (2017). Bile Acid Physiology. Ann. Hepatol. 16, S4–S14. 10.5604/01.3001.0010.5493 29080336

[B7] Dixon S. L. Smondyrev A. M. Knoll E. H. Rao S. N. Shaw D. E. Friesner R. A. (2006). PHASE: a New Engine for Pharmacophore Perception, 3D QSAR Model Development, and 3D Database Screening: 1. Methodology and Preliminary Results. J. Comput. Aided Mol. Des. 20 (10-11), 647–671. 10.1007/s10822-006-9087-6 17124629

[B8] Döring B. Lütteke T. Geyer J. Petzinger E. (2012). The SLC10 Carrier Family. Curr. Top. Membr. 70, 105–168. 10.1016/B978-0-12-394316-3.00004-1 23177985

[B9] Drexler J. F. Geipel A. König A. Corman V. M. van Riel D. Leijten L. M. (2013). Bats Carry Pathogenic Hepadnaviruses Antigenically Related to Hepatitis B Virus and Capable of Infecting Human Hepatocytes. Proc. Natl. Acad. Sci. 110 (40), 16151–16156. 10.1073/pnas.1308049110 24043818 PMC3791787

[B10] Fernandes C. F. Godoy J. R. Döring B. Cavalcanti M. C. O. Bergmann M. Petzinger E. (2007). The Novel Putative Bile Acid Transporter SLC10A5 Is Highly Expressed in Liver and Kidney. Biochem. Biophys. Res. Commun. 361 (1), 26–32. 10.1016/j.bbrc.2007.06.160 17632081

[B11] Fietz D. Bakhaus K. Wapelhorst B. Grosser G. Günther S. Alber J. (2013). Membrane Transporters for Sulfated Steroids in the Human Testis - Cellular Localization, Expression Pattern and Functional Analysis. PLoS One 8 (5), e62638. 10.1371/journal.pone.0062638 23667501 PMC3648580

[B12] Fukano K. Tsukuda S. Watashi K. Wakita T. (2019). Concept of Viral Inhibitors via NTCP. Semin. Liver Dis. 39 (1), 078–085. 10.1055/s-0038-1676804 30809790

[B15] Geyer J. Bakhaus K. Bernhardt R. Blaschka C. Dezhkam Y. Fietz D. (2017). The Role of Sulfated Steroid Hormones in Reproductive Processes. J. Steroid Biochem. Mol. Biol. 172, 207–221. 10.1016/j.jsbmb.2016.07.002 27392637

[B14] Geyer J. Döring B. Meerkamp K. Ugele B. Bakhiya N. Fernandes C. F. (2007). Cloning and Functional Characterization of Human Sodium-dependent Organic Anion Transporter (SLC10A6). J. Biol. Chem. 282 (27), 19728–19741. 10.1074/jbc.M702663200 17491011

[B13] Geyer J. Wilke T. Petzinger E. (2006). The Solute Carrier Family SLC10: More Than a Family of Bile Acid Transporters Regarding Function and Phylogenetic Relationships. Naunyn Schmied Arch. Pharmacol. 372 (6), 413–431. 10.1007/s00210-006-0043-8 16541252

[B16] Godoy J. R. Fernandes C. Döring B. Beuerlein K. Petzinger E. Geyer J. (2007). Molecular and Phylogenetic Characterization of a Novel Putative Membrane Transporter (SLC10A7), Conserved in Vertebrates and Bacteria. Eur. J. Cell Biol. 86 (8), 445–460. 10.1016/j.ejcb.2007.06.001 17628207

[B19] Grosser G. Bennien J. Sánchez-Guijo A. Bakhaus K. Döring B. Hartmann M. (2018). Transport of Steroid 3-sulfates and Steroid 17-sulfates by the Sodium-dependent Organic Anion Transporter SOAT (SLC10A6). J. Steroid Biochem. Mol. Biol. 179, 20–25. 10.1016/j.jsbmb.2017.09.013 28951227

[B18] Grosser G. Döring B. Ugele B. Geyer J. Kulling S. E. Soukup S. T. (2015). Transport of the Soy Isoflavone Daidzein and its Conjugative Metabolites by the Carriers SOAT, NTCP, OAT4, and OATP2B1. Arch. Toxicol. 89 (12), 2253–2263. 10.1007/s00204-014-1379-3 25319728

[B17] Grosser G. Fietz D. Günther S. Bakhaus K. Schweigmann H. Ugele B. (2013). Cloning and Functional Characterization of the Mouse Sodium-dependent Organic Anion Transporter Soat (Slc10a6). J. Steroid Biochem. Mol. Biol. 138, 90–99. 10.1016/j.jsbmb.2013.03.009 23562556

[B20] Hagenbuch B. Meier P. J. (1994). Molecular Cloning, Chromosomal Localization, and Functional Characterization of a Human Liver Na+/bile Acid Cotransporter. J. Clin. Invest. 93 (3), 1326–1331. 10.1172/JCI117091 8132774 PMC294097

[B21] Hagenbuch B. Meier P. (1996). Sinusoidal (Basolateral) Bile Salt Uptake Systems of Hepatocytes. Semin. Liver Dis. 16 (2), 129–136. 10.1055/s-2007-1007226 8781018

[B22] Hu N.-J. Iwata S. Cameron A. D. Drew D. (2011). Crystal Structure of a Bacterial Homologue of the Bile Acid Sodium Symporter ASBT. Nature 478 (7369), 408–411. 10.1038/nature10450 21976025 PMC3198845

[B23] Iwamoto M. Saso W. Sugiyama R. Ishii K. Ohki M. Nagamori S. (2019). Epidermal Growth Factor Receptor Is a Host-Entry Cofactor Triggering Hepatitis B Virus Internalization. Proc. Natl. Acad. Sci. USA 116, 8487–8492. 10.1073/pnas.1811064116 30952782 PMC6486715

[B25] Karakus E. Wannowius M. Müller S. F. Leiting S. Leidolf R. Noppes S. (2020). The Orphan Solute Carrier SLC10A7 Is a Novel Negative Regulator of Intracellular Calcium Signaling. Sci. Rep. 10 (1), 7248. 10.1038/s41598-020-64006-3 32350310 PMC7190670

[B24] Karakus E. Zahner D. Grosser G. Leidolf R. Gundogdu C. Sánchez-Guijo A. (2018). Estrone-3-Sulfate Stimulates the Proliferation of T47D Breast Cancer Cells Stably Transfected with the Sodium-Dependent Organic Anion Transporter SOAT (SLC10A6). Front. Pharmacol. 9, 941. 10.3389/fphar.2018.00941 30186172 PMC6111516

[B26] Karpen S. J. Kelly D. Mack C. Stein P. (2020). Ileal Bile Acid Transporter Inhibition as an Anticholestatic Therapeutic Target in Biliary Atresia and Other Cholestatic Disorders. Hepatol. Int. 14 (5), 677–689. 10.1007/s12072-020-10070-w 32653991

[B27] Kirstgen M. Lowjaga K. A. A. T. Müller S. F. Goldmann N. Lehmann F. Alakurtti S. (2020). Selective Hepatitis B and D Virus Entry Inhibitors from the Group of Pentacyclic Lupane-type Betulin-Derived Triterpenoids. Sci. Rep. 10 (1), 21772. 10.1038/s41598-020-78618-2 33303817 PMC7729925

[B28] Koepsell H. (2019). Multiple Binding Sites in Organic Cation Transporters Require Sophisticated Procedures to Identify Interactions of Novel Drugs. Biol. Chem. 400 (2), 195–207. 10.1515/hsz-2018-0191 30138103

[B29] König A. Döring B. Mohr C. Geipel A. Geyer J. Glebe D. (2014). Kinetics of the Bile Acid Transporter and Hepatitis B Virus Receptor Na+/taurocholate Cotransporting Polypeptide (NTCP) in Hepatocytes. J. Hepatol. 61 (4), 867–875. 10.1016/j.jhep.2014.05.018 24845614

[B30] Kramer W. Glombik H. (2006). Bile Acid Reabsorption Inhibitors (BARI): Novel Hypolipidemic Drugs. Curr. Med. Chem. 13 (9), 997–1016. 10.2174/092986706776361003 16611081

[B31] Li X. Liu H. Cheng W. Wang J. Zhang H. Lu F. (2020). Junceellolide B, a Novel Inhibitor of Hepatitis B Virus. Bioorg. Med. Chem. 28 (16), 115603. 10.1016/j.bmc.2020.115603 32690259

[B32] Lowjaga K. A. A. T. Kirstgen M. Müller S. F. Goldmann N. Lehmann F. Glebe D. (2021). Long-term Trans-inhibition of the Hepatitis B and D Virus Receptor NTCP by Taurolithocholic Acid. Am. J. Physiol.-Gastrointestinal Liver Physiol. 320 (1), G66–G80. 10.1152/ajpgi.00263.2020 33174454

[B33] Müller S. F. König A. Döring B. Glebe D. Geyer J. (2018). Characterisation of the Hepatitis B Virus Cross-Species Transmission Pattern via Na+/taurocholate Co-transporting Polypeptides from 11 New World and Old World Primate Species. PLoS One 13 (6), e0199200. 10.1371/journal.pone.0199200 29912972 PMC6005513

[B34] Ni Y. Lempp F. A. Mehrle S. Nkongolo S. Kaufman C. Fälth M. (2014). Hepatitis B and D Viruses Exploit Sodium Taurocholate Co-transporting Polypeptide for Species-specific Entry into Hepatocytes. Gastroenterology 146 (4), 1070–1083. 10.1053/j.gastro.2013.12.024 24361467

[B35] Ruggiero M. J. Malhotra S. Fenton A. W. Swint-Kruse L. Karanicolas J. Hagenbuch B. (2021). A Clinically Relevant Polymorphism in the Na+/taurocholate Cotransporting Polypeptide (NTCP) Occurs at a Rheostat Position. J. Biol. Chem. 296, 100047. 10.1074/jbc.RA120.014889 PMC7948949 33168628

[B36] Shneider B. L. Dawson P. A. Christie D. M. Hardikar W. Wong M. H. Suchy F. J. (1995). Cloning and Molecular Characterization of the Ontogeny of a Rat Ileal Sodium-dependent Bile Acid Transporter. J. Clin. Invest. 95 (2), 745–754. 10.1172/JCI117722 7860756 PMC295543

[B37] Stieger B. Hagenbuch B. Landmann L. Höchli M. Schroeder A. Meier P. J. (1994). *In situ* Localization of the Hepatocytic Na+/taurocholate Cotransporting Polypeptide in Rat Liver. Gastroenterology 107 (6), 1781–1787. 10.1016/0016-5085(94)90821-4 7958692

[B38] Urso A. D’Ovidio F. Xu D. Emala C. W. Bunnett N. W. Perez-Zoghbi J. F. (2020). Bile Acids Inhibit Cholinergic Constriction in Proximal and Peripheral Airways from Humans and Rodents. Am. J. Physiol.-Lung Cell Mol. Physiol. 318 (2), L264–L275. 10.1152/ajplung.00242.2019 31800261 PMC7474253

[B39] Wang X. Lyu Y. Ji Y. Sun Z. Zhou X. (2021). An Engineered Disulfide Bridge Traps and Validates an Outward-Facing Conformation in a Bile Acid Transporter. Acta Cryst. Sect D Struct. Biol. 77 (Pt 1), 108–116. 10.1107/S205979832001517X 33404530

[B40] Weinman S. A. (1997). Electrogenicity of Na(+)-Coupled Bile Acid Transporters. Yale J. Biol. Med. 70 (4), 331–340. 9626753 PMC2589348

[B41] Wettengel J. M. Burwitz B. J. (2020). Innovative HBV Animal Models Based on the Entry Receptor NTCP. Viruses 12 (8), 828. 10.3390/v12080828 PMC7472226 32751581

[B42] Wong M. H. Rao P. N. Pettenati M. J. Dawson P. A. (1996). Localization of the Ileal Sodium-Bile Acid Cotransporter Gene (SLC10A2) to Human Chromosome 13q33. Genomics 33 (3), 538–540. 10.1006/geno.1996.0233 8661017

[B43] Yan H. Zhong G. Xu G. He W. Jing Z. Gao Z. (2012). Sodium Taurocholate Cotransporting Polypeptide Is a Functional Receptor for Human Hepatitis B and D Virus. eLife 1, 1. 10.7554/eLife.00049 PMC3485615 23150796

[B44] Zhou X. Levin E. J. Pan Y. McCoy J. G. Sharma R. Kloss B. (2014). Structural Basis of the Alternating-Access Mechanism in a Bile Acid Transporter. Nature 505 (7484), 569–573. 10.1038/nature12811 24317697 PMC4142352

