# Supplementary material for: Substrate Specificities and Inhibition Pattern of the Solute Carrier Family 10 Members NTCP, ASBT and SOAT
Source: Front Mol Biosci. 2021 May 17;8:689757. doi: 10.3389/fmolb.2021.689757 (PMC8165160; doi:10.3389/fmolb.2021.689757)
Supplement: Supplementary file 1 [file DataSheet1.PDF]

Substrate specificities and inhibition pattern of the Solute Carrier Family 10 members NTCP, ASBT and SOAT

Gary Grosser[†], Simon Franz Müller[†], Michael Kirstgen, Barbara Döring, Joachim Geyer[*]

Institute of Pharmacology and Toxicology, Faculty of Veterinary Medicine, Justus Liebig University Giessen, Biomedical Research Center Seltersberg (BFS), Schubertstr. 81, 35392 Giessen

[†]These authors have contributed equally to this work and share first authorship.

Supplementary Figure 1

NTCP, ASBT, SOAT and their place within the SLC10 carrier family

The SLC10 family can be subdivided in three groups with two homologous carriers each (Fig. 1A). The first subgroup contains the carriers ASBT and SOAT, which have a pairwise amino acid identity of 47.9%, being the overall highest sequence homology within the SLC10 family. Subgroup two consists of NTCP and the neuronal carrier SLC10A4. Based on this, NTCP is phylogenetically quite distant to ASBT and SOAT with amino acid sequence identities of only 39.0% and 38.4%, respectively (Fig. 1B). As NTCP and ASBT are well established BA carriers, but SOAT transports sulfated steroid hormones, but not BAs, the phylogenetic relationship of these carriers is not in line with their functional properties. An additional SLC10 subgroup consists of the orphan carriers SLC10A3 and SLC10A5, which have not shown BA transport activity yet (Fig. 1A). Compared to the carriers SLC10A1-SLC10A6, SLC10A7 is much more distant. As SLC10A7 recently was identified as a negative regulator of intracellular calcium signaling (RCAS), its function is clearly different from NTCP, ASBT and SOAT. In addition to the eukaryotic proteins SLC10A1-SLC10A7, two bacterial carrier proteins were added to the phylogenetic analysis, being the so-called bacterial Asbts from *Neisseria meningitidis* ($Asbt_{Nm}$) and from *Yersinia frederiksenii* ($Asbt_{Yf}$). However, as these bacterial proteins are equidistant to NTCP, ASBT, and SOAT, they should be more considered as bacterial SLC10 homologous rather than as bacterial Asbt orthologues. They were integrated to show their similarity to the SLC10 family as they served as templates for homology modelling of NTCP, ASBT and SOAT. For comparative transport studies stably transfected HEK293 cells were generated based on the identical HEK293-FlpIn cell line and following the identical protocol. The generated cell lines NTCP-HEK293, ASBT-HEK293, and SOAT-HEK293 showed significant overexpression of the respective carrier as shown by comparative quantitative expression analysis (Fig. 1C).

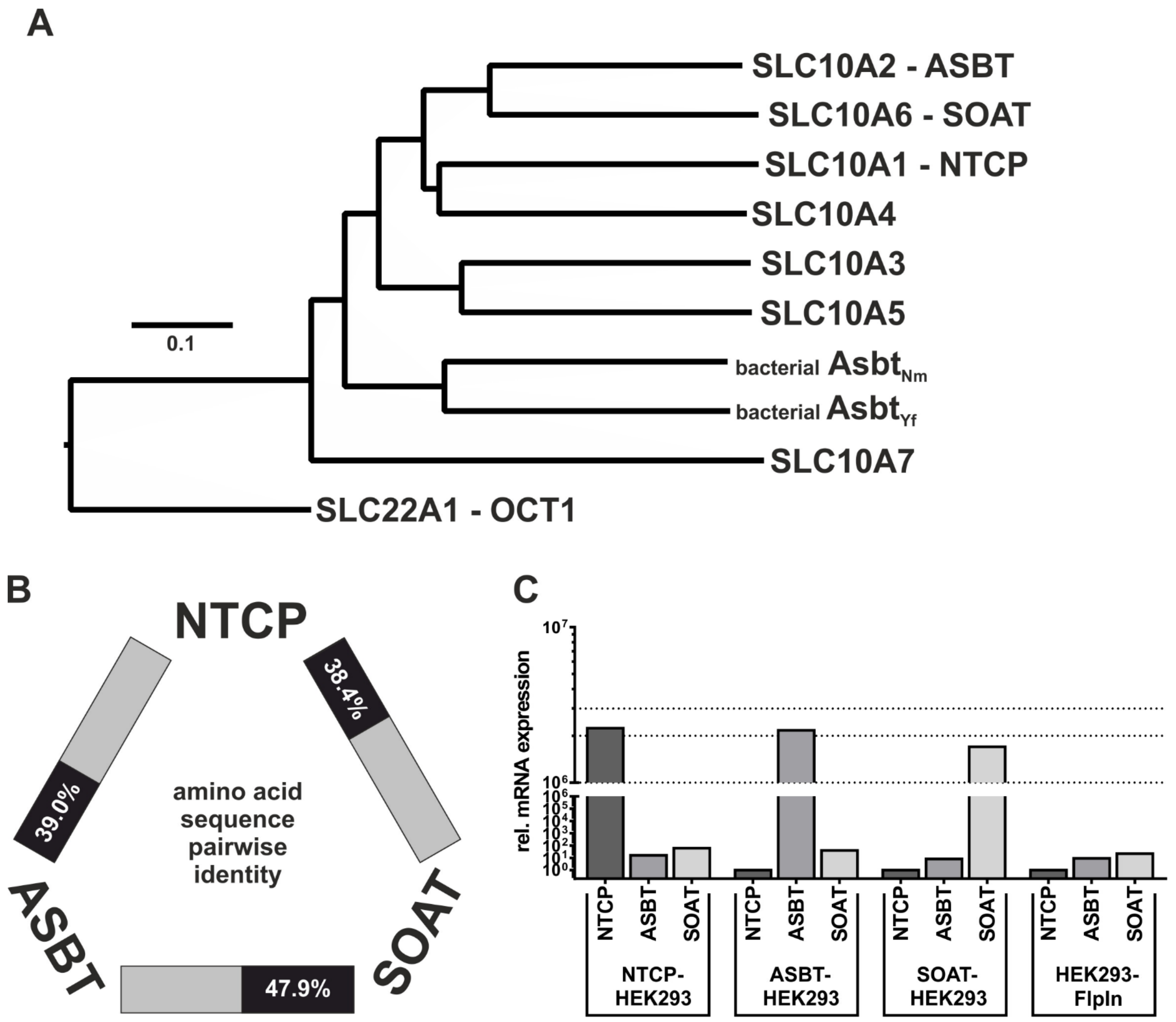

Supplementary Fig. 1: Comparison of the SLC10 carriers NTCP, ASBT and SOAT. **(A)** Dendrogram of the human SLC10 carriers SLC10A1-SLC10A7 and the bacterial proteins $Asbt_{Nm}$ (from *Neisseria meningitidis*) and $Asbt_{Yf}$ (from *Yersinia frederiksenii*). Human SLC22A1 (Organic Cation Transporter 1) was used as outgroup. Scale bar represents 0.1 amino acid changes per site on horizontal distance. **(B)** Amino acid sequence identity between the carriers NTCP, ASBT and SOAT, based on pairwise sequence alignment with the ClustalW algorithm. **(C)** Carrier mRNA expression of the stably transfected cell lines NTCP-HEK293, ASBT-HEK293, and SOAT-HEK293 as well as of the original cell line HEK293-FlpIn. The mRNA expression was analyzed by quantitative real-time PCR with cDNA from the indicated tetracycline-induced stable cell lines. Relative carrier expression was calculated by the $2^{-\Delta\Delta CT}$ transformation and represents carrier expression x-times higher compared with the calibrator (NTCP expression in SOAT-HEK293 cells). ACTB served as endogenous control. Values represent means of duplicate determinations.
